# Changes in ware–decoration associations in ceramic assemblages from Wacheqsa, Chavín de Huántar, Peru

**DOI:** 10.1371/journal.pone.0361889

**Published:** 2026-10-29

**Authors:** Christian Mesía-Montenegro

**Affiliations:** Dirección de Investigación e Innovación, Universidad Privada del Norte, Lima, Peru; Maria Curie-Sklodowska University: Uniwersytet Marii Curie-Sklodowskiej, POLAND

## Abstract

Ceramic differentiation can involve altered associations among continuing attributes without wholesale categorical replacement. At Wacheqsa, Chavín de Huántar, Peru, this study compares ware composition, decorative techniques, and their associations in 1,308 decorated or surface-treated sherd observations from five archaeological contexts. Early Platforms and Water Flood constitute the earlier Urabarroid aggregate; Stone Rooms, Midden, and Late Platforms constitute the later Janabarroid aggregate. These labels describe ceramic affinities within pre-existing context sets, whose stratigraphic networks are only partly connected and whose formation histories differ. On identical common support of nine wares and six techniques (n = 334/822), ware-margin Hellinger distance is 0.430 (95% fixed-count interval 0.392–0.481), technique-margin distance is 0.588 (0.551–0.634), and joint distance is 0.734 (0.714–0.793). A homogeneous-association model rejects a common ware–technique association after aggregate-specific margins are fitted (G^2^ = 118.59; pboot = 0.0001); the result persists without Midden (G^2^ = 90.16; pboot = 0.0001). Early Platforms differs from each later context, and stamping/impression is prominent in all three later samples, although those contexts retain distinct joint profiles. Nine of eleven ware categories occur in both aggregates, with pronounced differences in the association of polished wares with incision and stamping/impression. The contrast is consistent with differing decorated repertoires across activity and depositional settings, including contexts associated with collective consumption. Local superpositions and the published chronology support a temporal interpretation, but the contribution of elapsed time cannot be separated from differences in activity and deposition.

## Introduction

Wacheqsa is a 1.4 ha sector immediately north of the monumental core of Chavín de Huántar (Fig 1). This study compares five depositional contexts: Early Platforms, Water Flood, Stone Rooms, Midden, and Late Platforms. Early Platforms and Water Flood constitute the earlier Urabarroid aggregate; the other three units constitute the later Janabarroid aggregate. These local affinity labels apply to context sets established during the original stratigraphic analysis. The comparison evaluates material differences across those settings rather than reconstructing a continuous five-stage sequence.

**Fig 1:**
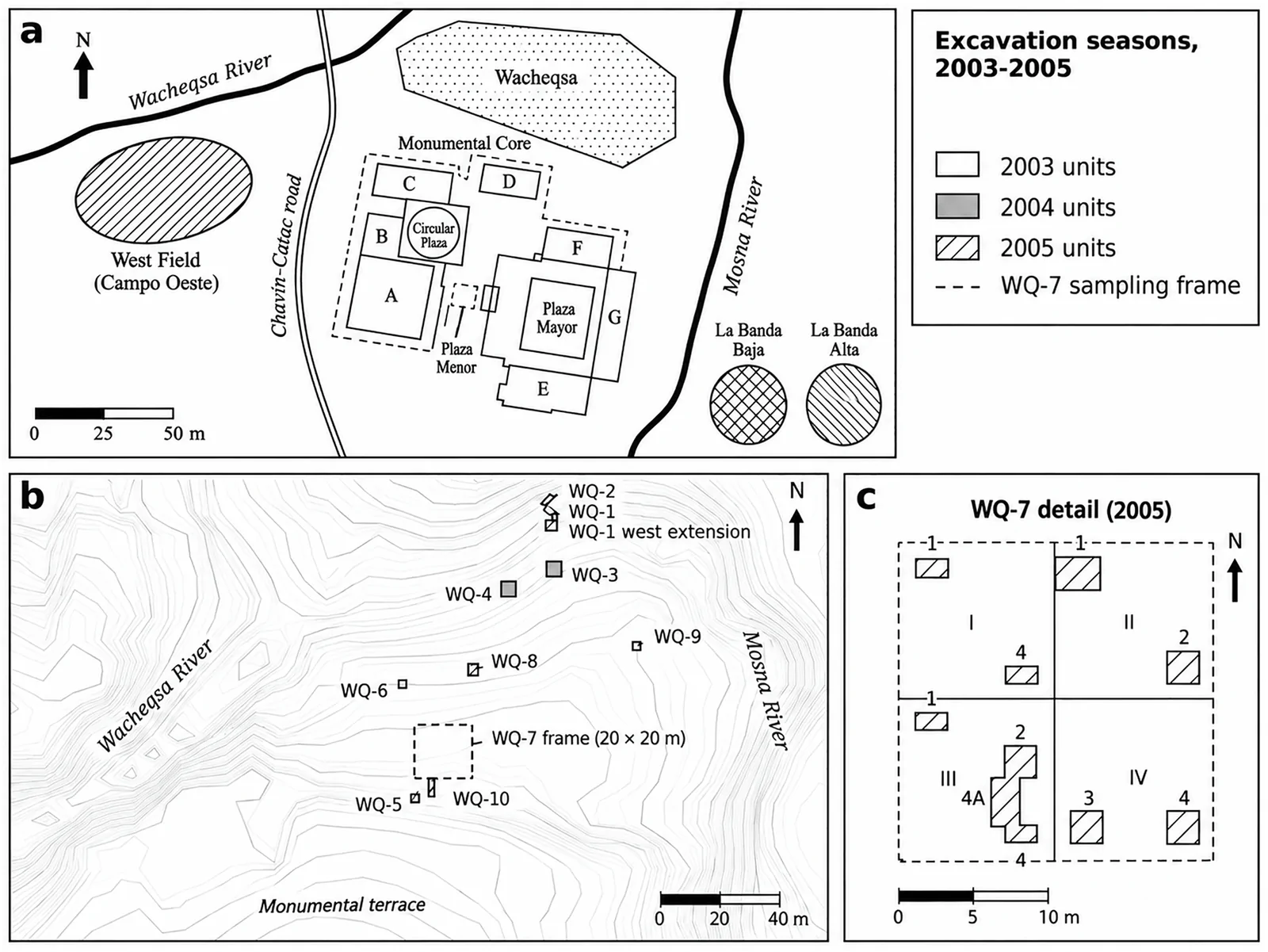
Schematic location and excavation maps of Wacheqsa. (a) Schematic location map showing Wacheqsa in relation to the monumental core, West Field (Campo Oeste), La Banda Baja, La Banda Alta, the Wacheqsa River, the Mosna River, and the Chavín–Catac road. Monumental architecture and sector boundaries are simplified from project base maps; the panel is intended to show spatial relationships rather than survey-level architectural detail. (b) Schematic topographic map of the Wacheqsa sector showing excavation units WQ-1–WQ-10 and the WQ-7 systematic sampling frame. Excavation-season symbols distinguish units excavated in 2003, 2004, and 2005. (c) Detail of the 2005 WQ-7 systematic sampling frame showing Sectors I–IV and the excavated subunits. Scale bars are shown in each panel.

Ceramic assemblages can differ in ware frequencies, technique frequencies, and the associations between them. Published ceramic types often combine color, finish, form, and decoration. Although useful for seriation and comparison, these constructs can obscure which attributes account for an assemblage contrast. At Wacheqsa, the question is whether the contrast involves categorical replacement, changes in the abundance of familiar attributes, or different combinations of continuing wares and techniques.

Assemblage-scale ceramic analysis offers a way to study sherd records when individual artifacts provide incomplete evidence of technology and use [1]. Ceramic seriation addresses a different problem: deriving relative order from assemblage frequencies, whose stability can be sensitive to small or multicomponent samples [2]. Here, the contextual framework precedes the frequency comparison. The contribution is to distinguish differences in the abundance of recorded attributes from differences in their association, and to determine whether the latter persist across Wacheqsa contexts with contrasting formation histories.

The distinction also addresses the risk of circularity. In this study, “type” refers only to historically published composite constructs such as Burger’s Urabarriu, Chakinani, and Janabarriu [3–5]. Those types are not independently coded variables in the Wacheqsa matrices, and reconstructing type assignments from the same surface attributes analyzed here would make a type-versus-attribute test circular. The non-decoration marginal comparison therefore uses the recorded ware categories, not a reconstructed Burger-type classification; decorative technique provides the second margin. Accordingly, the aim is not to reclassify Wacheqsa sherds into Burger’s types or to validate a received seriation, but to determine how ceramic composition and ware–technique association differ among archaeological context sets established independently of the present frequency analysis.

The three primary comparisons distinguish these possibilities. Ware margins describe the relative abundance of recorded color-plus-surface-finish categories; technique margins describe decorative frequencies. The joint table describes how techniques are distributed among wares. Testing association after fitting both margins evaluates whether differences in those pairings extend beyond differences in either attribute alone.

Hellinger distance provides comparable effect-size summaries for the ware margin, technique margin, and joint table [6]. A boundary-aware homogeneous-association model then asks whether ware × technique association remains common after aggregate-specific ware and technique margins are fitted [7]. Residuals and correspondence analysis localize structure, but none of these statistics identifies the social or behavioral mechanism that produced it.

The observations are classified sherds, potentially including several fragments from one vessel [8]. They document the sampled assemblages rather than reconstructed vessel populations. The analysis identifies material differences to be considered within the documented Wacheqsa sequence [9].

The primary analysis compares ware margins, technique margins, and joint composition on identical shared support, then tests association heterogeneity after fitting both margins. Removing Midden assesses the influence of the largest later sample. Comparisons among the three later units and cellwise diagnostics locate contextual and attribute-level differences.

## Materials and methods

### Archaeological context and analytical units

#### Wacheqsa stratigraphy.

The sector lies between the Wacheqsa and Mosna rivers. A landslide in 1945 substantially altered its surface, making excavated stratigraphy the principal evidence for the Formative occupation [9,10].

Fieldwork between 2003 and 2005 combined purposive exposures with systematic excavation in a 20 × 20 m block in the south-central sector (Fig 2). In total, 132 m^2^ were excavated. Deposits were recorded as three-dimensional surfaces, related through Harris matrices, and characterized by stratigraphic position, geometry, matrix, architectural association, artifact content, and chronology [9]. Detailed excavation inventories and densities are summarized in Table 1 rather than used as sampling fractions.

**Table 1:** Archaeological units, contextual evidence, and interpretations in the Wacheqsa sequence.

Analytical unit	Aggregate	Formation and stratigraphy	Excavated inventory	Contextual interpretation
Early Platforms	Earlier / Urabarroid	Low-density platform surfaces beneath Stone Rooms	20.3 m^3^; 978 artifacts; ≈ 48/m^3^	Small neckless jars and small-to-medium bowls; domestic/provisioning signal
Water Flood	Earlier / Urabarroid	At least three major water-flow episodes plus smaller events	5.72 m^3^; 330 artifacts; ≈ 58/m^3^	Water-related formation; demonstrates heterogeneity within earlier aggregate
Stone Rooms	Later / Janabarroid	Rooms and alleys above Early Platforms; cleaned, filled, and sealed	22.3 m^3^; 3,425 artifacts; ≈ 154/m^3^	Segmented activity space; larger serving forms; broader special-material inventory
Midden	Later / Janabarroid	44 strata; three major refuse episodes plus smaller accumulations	22.03 m^3^; 15,814 fragments; 184.2 kg animal bone	Recurrent collective consumption, cleaning, and secondary disposal
Late Platforms	Later / Janabarroid	Compact, stony, low-density fills associated with closure	5.88 m^3^; 102 artifacts; 17.3/m^3^	Low-density closure/reorganization; not functionally equivalent to Early Platforms

Note: The analytical matrices contain a separate analytical corpus: Early Platforms n = 330, Water Flood n = 22, Stone Rooms n = 215, Midden n = 691, and Late Platforms n = 50. Excavation densities and analytical counts use different selection and counting procedures and cannot be converted into sampling fractions.

**Fig 2:**
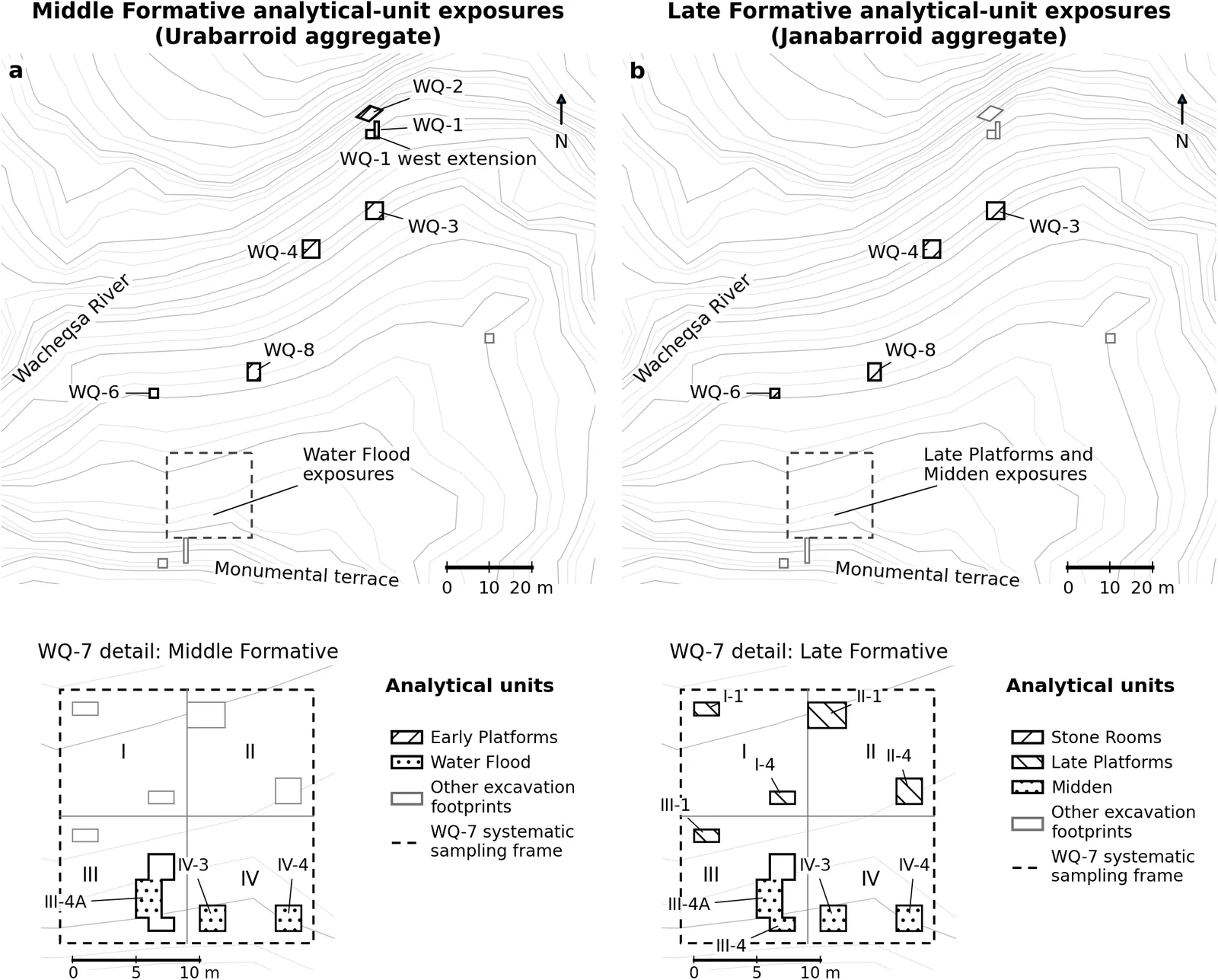
Documented excavation exposures of the Wacheqsa analytical units. (a) Middle Formative contexts associated with the Urabarroid aggregate: Early Platforms and Water Flood. (b) Late Formative contexts associated with the Janabarroid aggregate: Stone Rooms, Late Platforms, and Midden. The principal maps identify excavation footprints in which deposits assigned to each analytical unit were documented; the lower plans show the relevant WQ-7 systematic-sampling subunits. Symbols represent observed excavated exposures rather than mutually exclusive surface territories or the complete modeled extents of the three-dimensional analytical units. Early Platforms and Stone Rooms overlap in plan because Stone Rooms overlie Early Platforms in several excavations. Water Flood and Midden similarly overlap at the southern end of WQ-7 because Midden deposits overlie the water-laid strata. The irregular Sector III footprint represents the contiguous expanded excavation comprising Units 2, 4A, and 4. Topographic contours and excavation geometry derive from the author’s CAD model.

The author compiled the context-level Harris matrix in Stratify in 2007. The five analytical units are higher-order groupings within this model, which contains parallel and partly connected depositional networks. They do not form a continuous, equally spaced five-stage sequence.

#### Construction of analytical units and aggregates.

The five analytical units were constructed during the original post-excavation analysis, before the ware-decoration frequency comparisons reported here. Individual deposits were distinguished in the field and organized into excavation-unit Harris matrices using superposition, original horizontality, and continuity. These matrices were subsequently merged in Stratify and integrated with three-dimensional CAD models of deposit location, geometry, and elevation. Deposits were grouped into analytical units according to stratigraphic position, spatial continuity, sediment matrix, geometry, architectural association, and inferred formation history. General artifact content and available chronological evidence also contributed to contextual characterization. The procedure was therefore independent of the standardized ware-decoration matrices analyzed in the present study, but it was not blind to all archaeological materials [9].

Each unit represents a distinct depositional configuration. Early Platforms comprises near-horizontal platform surfaces, floors, and associated low-density fills. Water Flood consists of correlated sequences of sand and gravel deposited through repeated water-flow events. Stone Rooms comprises rooms, alleys, floors, cleaning episodes, and subsequent fills constructed above Early Platforms in several exposures. Midden consists of repeated high-density refuse deposits that overlie or cut water-related strata. Late Platforms comprises compact, low-density stone and cobble fills associated with later construction and the sealing or reorganization of portions of the room-and-alley system. These units are differentiated depositional and activity contexts rather than interchangeable or equally spaced chronological replicates.

The earlier–later grouping combines local superposition with correlations between excavation exposures. Stone Rooms overlies Early Platforms in several exposures. The original stratigraphic synthesis places Early Platforms layer 6 in WQ-4 below the Stone Rooms architectural network, with an intervening deposit, and correlates that network with the context represented by WQ-8 layer 3 (section 7.2.3.1, p. 151) [9]. The relationship between the WQ-4 and WQ-8 contexts therefore depends on cross-exposure correlation rather than direct superposition of those two contexts. Midden overlies or cuts Water Flood in southern WQ-7, providing a separate local earlier–later relationship. Early Platforms contains no Janabarroid material, whereas Late Platforms does. Late Platforms also includes a deeper construction deposit associated with radiocarbon determination AA75388, whose early age and possible overlap with Early Platforms were noted in the original analysis (section 7.2.3.5, p. 153) [9]. The Janabarroid material supports the assignment of Late Platforms to the later ceramic aggregate, although the early determination cautions against assuming that every deposit in this unit postdates Early Platforms. Ceramic affinities motivate the aggregate names; the present frequency analysis does not independently establish their ages.

Basal contexts contain predominantly Urabarroid materials; Stone Rooms, Midden, and Late Platforms deposits contain predominantly Janabarroid materials. These affinities explain the aggregate labels. The context sets and their principal relative order were established before the present frequency analysis.

### Local terminology and chronological scope

#### Urabarroid and Janabarroid.

Wacheqsa was occupied broadly between approximately 1100 and 550 cal BCE [9–11]. The ten conventional radiocarbon dates and their analytical-unit assignments are published in Mesía-Montenegro [10], Table 2 (p. 887); Fig 3 on that page plots calibrated dates rather than modeled aggregate boundaries. The chronological estimates reported here are taken from Mesía-Montenegro and Weber [12], Table 1 (p. 782); the published Bayesian model is shown in Fig 3 (p. 781). End Urabarroid–Start Janabarroid is 878–806 cal BCE and End Janabarroid is 760–581 cal BCE, both at 68.3% probability. Table 2 combines the conventional ages from the 2022 paper, the modeled intervals from the 2024 paper, and the excavation contexts from the dissertation. These published posterior estimates describe the aggregate chronology; they do not directly date ceramic production or the change in decorative practice. No new calibration or Bayesian model is reported here.

**Table 2:** Published radiocarbon dates and modeled ranges for the Wacheqsa sequence.

Laboratory code	Analytical unit	14C age BP ± 1σ	Modeled cal BCE	Sampled context
AA75387	Early Platforms	2580 ± 36	896–815	WQ-4, layer 6
AA75386	Early Platforms	2664 ± 35	901–820	WQ-2, floor 3 / layer 6
AA75383	Water Flood	2679 ± 35	902–823	WQ-7, Sector IV, Unit 4, layer 20
AA75385	Early Platforms	2769 ± 35	931–852	WQ-1, layer 5
GX31647	Early Platforms	2880 ± 40	992–899	WQ-6, Feature 3
AA75382	Midden	2481 ± 35	775–686	WQ-7, Sector IV, Unit 4, layer 10
AA75390	Stone Rooms	2500 ± 35	780–668	WQ-8, layer 3
AA75384	Midden	2512 ± 35	787–668	WQ-7, Sector IV, Unit 4, layer 14
AA75389	Midden	2573 ± 34	801–766	WQ-7, Sector III, Unit 4, layer 8
AA75388	Late Platforms	2740 ± 37	847–803	WQ-7, Sector III, Unit 1, layer 9

Sources: conventional ages and analytical-unit assignments from Mesía-Montenegro [10], Table 2, p. 887; modeled intervals from Mesía-Montenegro and Weber [12], Table 1, p. 782; excavation contexts from Mesía-Montenegro [9], sections 7.2.3.1–7.2.3.5, pp. 150–153. All modeled intervals are the published 68.3% estimates. Published boundaries are Start Urabarroid 1023–904, End Urabarroid–Start Janabarroid 878–806, and End Janabarroid 760–581 cal BCE.

**Fig 3:**
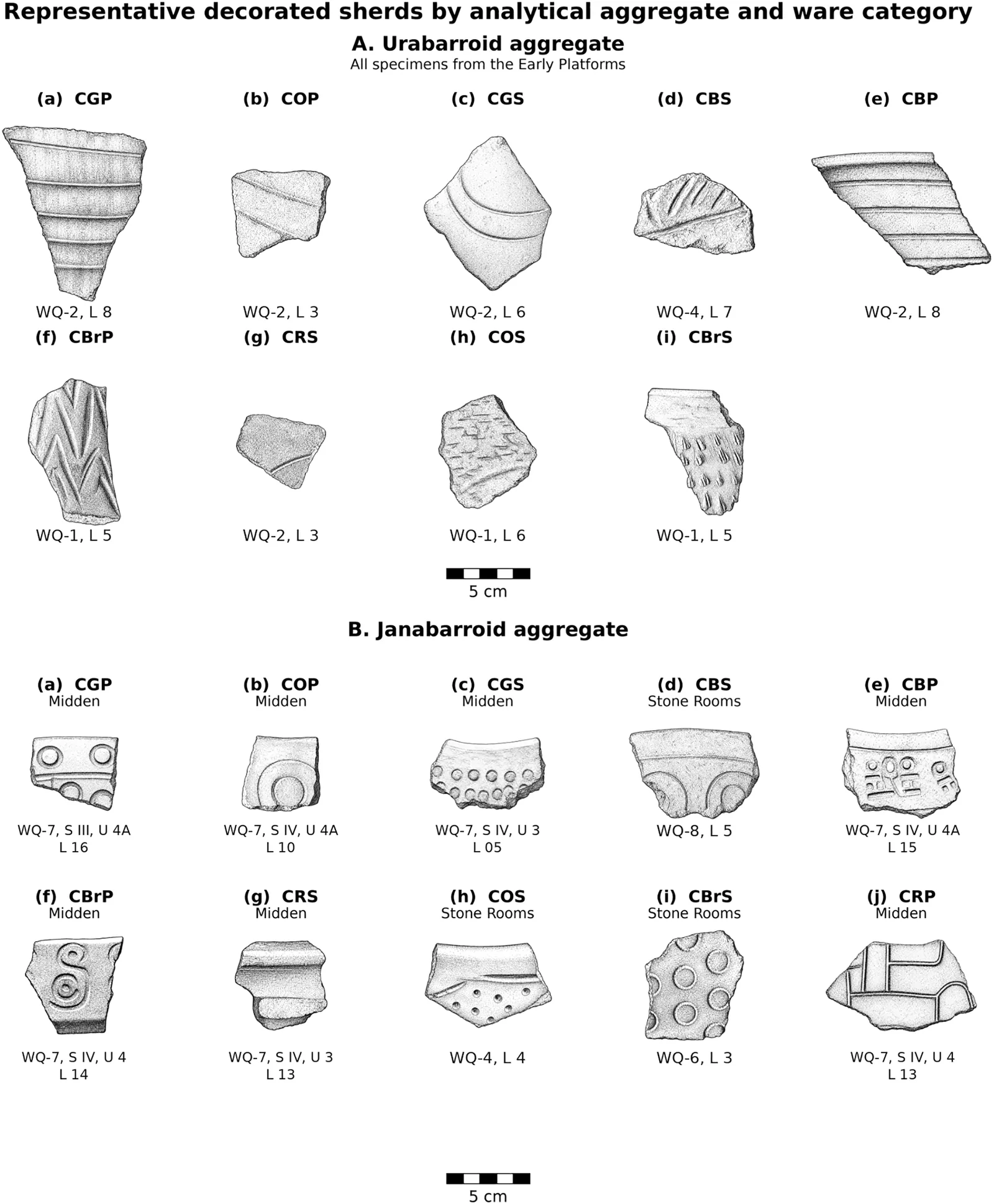
Representative decorated sherds by analytical aggregate, ware category, and excavation provenience. (A) Urabarroid aggregate (Early Platforms): (a) Chavín Gray Polished (CGP), WQ-2, Level 8; (b) Chavín Orange Polished (COP), WQ-2, Level 3; (c) Chavín Gray Smoothed (CGS), WQ-2, Level 6; (d) Chavín Black Smoothed (CBS), WQ-4, Level 7; (e) Chavín Black Polished (CBP), WQ-2, Level 8; (f) Chavín Brown Polished (CBrP), WQ-1, Level 5; (g) Chavín Red Slipped (CRS), WQ-2, Level 3; (h) Chavín Orange Smoothed (COS), WQ-1, Level 6; and (i) Chavín Brown Smoothed (CBrS), WQ-1, Level 5. (B) Janabarroid aggregate: (a) CGP, WQ-7, Sector III, Unit 4A, Level 16; (b) COP, WQ-7, Sector IV, Unit 4A, Level 10; (c) CGS, WQ-7, Sector IV, Unit 3, Level 05; (d) CBS, WQ-8, Level 5; (e) CBP, WQ-7, Sector IV, Unit 4A, Level 15; (f) CBrP, WQ-7, Sector IV, Unit 4, Level 14; (g) CRS, WQ-7, Sector IV, Unit 3, Level 13; (h) COS, WQ-4, Level 4; (i) CBrS, WQ-6, Level 3; and (j) Chavín Red Polished (CRP), WQ-7, Sector IV, Unit 4, Level 13. All specimens in panel A derive from Early Platforms. In panel B, specimens (a–c, e–g, j) derive from Midden, and specimens (d, h, i) from Stone Rooms. The labels identify recorded excavation proveniences, not unique specimens or museum inventory numbers. The archaeological collection is curated at the Museo Nacional de Chavín, Ancash, Peru. Individual inventory numbers are unavailable in the analytical records. The illustrated specimens can be traced to their excavation proveniences, although identifying individual objects would require consulting the original collection records at the Museo Nacional de Chavín. Scale bars represent 5 cm.

Urabarroid and Janabarroid denote local resemblance to the ceramic attribute complexes described by Burger [3–5]. The labels do not equate the Wacheqsa context sets with his calendar phase boundaries or imply common production or social identity.

#### The legacy sequence.

Burger’s Urabarriu-Chakinani-Janabarriu sequence derives from 12 residential excavations west of the Mosna River [5] and remains an important comparative framework. Its application as a site-wide chronological tool nevertheless requires caution because ceramic affinity, deposit assignment, and calendar inference are not fully independent, and broader correlations extend beyond the residential stratigraphic relationships on which the sequence was originally based.

The status of Chakinani as a discrete chronological phase remains unresolved. The four associated faunal determinations derive from different layers within a single thick deposit [5]. They constrain the ages of the sampled remains, but do not by themselves establish a distinct ceramic interval across Chavín de Huántar. The Wacheqsa analysis requires neither an intermediate Chakinani assemblage nor independently tests its existence. Treating Chakinani as a site-wide phase therefore requires evidence that distinguishes a temporally bounded ceramic assemblage from variation attributable to activity, deposition, and ceramic classification.

Cross-sector chronology must therefore be evaluated independently from ceramic resemblance. Architectural and multi-sector radiocarbon evidence provides additional constraints [11,13–16], while calibration structure, sample association, and model specification limit the precision with which radiocarbon-based phase boundaries can be defined [17–19]. For the present analysis, the legacy sequence supplies terminology and comparative background rather than an independent chronological framework.

#### Spatial scope.

Wacheqsa is a functionally and depositionally distinctive sector. Its profiles should be compared with independently documented assemblages elsewhere at Chavín rather than extended as a uniform site-wide trajectory.

### Analytical corpus and classification

#### Sampling and analytical corpus.

The finalized matrices contain 1,308 decorated or surface-treated sherd observations: 352 in the Urabarroid aggregate and 956 in the Janabarroid aggregate. Sherds were selected by simple random sampling without replacement, separately within each of the five analytical units. Each unit therefore constitutes a distinct analytical sample, and the aggregate matrices were generated by cellwise summation of the corresponding unit matrices. The realized unit totals are Early Platforms n = 330, Water Flood n = 22, Stone Rooms n = 215, Midden n = 691, and Late Platforms n = 50.

Under the random sampling design, sample proportions estimate the composition of the eligible sherd collection within each analytical unit. Collection totals are not preserved, so sampling fractions and finite-population corrections cannot be calculated, and pooled aggregates cannot be weighted by original collection sizes. Systematic refitting was not undertaken or is not retained in the analytical record, so vessel-level independence cannot be established.

#### Ware, decoration, and normalization.

After normalization, the Urabarroid detailed matrix contains 19 decoration categories and ten wares; the Janabarroid matrix contains 29 categories and ten wares. Nine wares occur in both: Chavín Gray Polished (CGP), Chavín Orange Polished (COP), Chavín Gray Smoothed (CGS), Chavín Black Smoothed (CBS), Chavín Black Polished (CBP), Chavín Brown Polished (CBrP), Chavín Red Slipped (CRS), Chavín Orange Smoothed (COS), and Chavín Brown Smoothed (CBrS). Bichrome occurs only in Urabarroid (n = 3), and Chavín Red Polished (CRP) only in Janabarroid (n = 68). The shared-ware detailed matrices contain 349 earlier and 888 later sherds (Fig 3).

Ware categories combine the recorded macroscopic color and surface finish. Detailed decoration is the single decorative or surface-treatment label assigned to each sherd; broad technique groups these labels through the preserved concordance. Type is reserved for published composite constructs such as Urabarriu, Chakinani, and Janabarriu [3–5].

The surviving matrices and concordance permit the analytical recoding to be checked. They do not preserve co-occurring treatments or document the original rule for selecting a single label when multiple treatments were present. Procedures for handling eroded or ambiguous specimens and observer-agreement assessments are also undocumented. The implications of these limitations are evaluated in the Discussion.

Standardized categories are aggregated to nine broad classes: appliqué, graphiting, incision, modeling, Other, perforation, punctation, slipping, and stamping/impression. “Other” retains heterogeneous labels—Eye motif, Floral motif, Mosna, Ofrendas, Pinched, Qotopukyo, and Wacheqsa—without asserting technological equivalence. Graphiting and slipping occur only in the earlier shared-ware table, whereas Other occurs only in the later table. The principal joint comparison therefore uses six classes represented in both aggregates: appliqué, incision, modeling, perforation, punctation, and stamping/impression. No post hoc reclassification of Pinched or other labels was introduced.

Because ware labels encode surface finish and the detailed treatment variable includes slipping, the full detailed matrices contain partial classificatory dependence. The detailed chi-square and residual analyses are therefore interpreted as descriptions of normalized classificatory structure rather than as relationships between wholly orthogonal attributes. Comparative inference rests principally on the shared-six technique set, which excludes slipping, graphiting, and aggregate-specific broad classes. Table 3 lists the nine shared wares and six broad techniques used in the primary common-support comparison (Table 4).

**Table 3:** Shared analytical categories used in the principal 9-ware × 6-technique common-support comparison.

Analytical dimension	Code/category	Operational label
Ware	CGP	Chavín Gray Polished
Ware	COP	Chavín Orange Polished
Ware	CGS	Chavín Gray Smoothed
Ware	CBS	Chavín Black Smoothed
Ware	CBP	Chavín Black Polished
Ware	CBrP	Chavín Brown Polished
Ware	CRS	Chavín Red Slipped
Ware	COS	Chavín Orange Smoothed
Ware	CBrS	Chavín Brown Smoothed
Broad technique	Appliqué	Appliqué
Broad technique	Incision	Incision
Broad technique	Modeling	Modeling
Broad technique	Perforation	Perforation
Broad technique	Punctation	Punctation
Broad technique	Stamping/impression	Stamping/impression

Note: Ware is the recorded macroscopic color-plus-surface-finish category. Broad technique is the analytical grouping of standardized decorative/surface-treatment labels. Bichrome and Chavín Red Polished are excluded from the principal common-support comparison because they occur in only one aggregate; graphiting, slipping, and Other are likewise excluded because they are not represented in both aggregates. The complete lexical concordance remains in S1 File.

**Table 4:** Reduction of the complete corpus to common support by mutually exclusive exclusion category.

Selection category	Urabarroid n	Janabarroid n
Complete corpus	352	956
Excluded ware only; technique retained	2	67
Excluded technique only; ware retained	15	66
Both ware and technique excluded	1	1
Total excluded	18	134
Common support retained	334	822
Excluded fraction (%)	5.1	14.0

Counts are recomputed from the five matrices and the preserved broad-technique concordance. Bichrome and Chavín Red Polished are the aggregate-specific wares; graphiting, slipping, and Other are excluded broad techniques. The mutually exclusive partition avoids double counting. In a sequential filter, ware exclusion removes 3 and 68 observations, followed by technique exclusion among retained wares removing 15 and 66. The complete corpus is 1,308 and the retained corpus is 1,156.

#### Analytical-unit composition.

Early Platforms contributes 330 of 352 Urabarroid observations (93.8%); Water Flood contributes 22 (6.3%). Midden contributes 691 of 956 Janabarroid observations (72.3%), Stone Rooms 215 (22.5%), and Late Platforms 50 (5.2%). These proportions characterize the analytical corpus, not prevalence in the original archaeological assemblages. The imbalance motivates explicit unit comparisons and a leave-Midden-out sensitivity analysis.

### Statistical analysis

#### Scope of inference.

The statistical analyses use conditional, model-based comparisons of finalized sherd counts. Random sampling supports estimation of proportions within each unit’s eligible collection, but missing collection totals prevent finite-population corrections and weighting by original collection size. The aggregate matrices sum the unit samples and are therefore weighted by their realized sample sizes. They do not estimate the composition of the combined eligible collections under known collection-size weights.

The leave-Midden-out analysis evaluates the sensitivity of the pooled aggregate comparison to the largest later unit sample; it does not remove confounding by chronology, activity composition, formation process, fragmentation, preservation, or recovery. The Poisson bootstrap used for log-linear calibration is model-based and does not reconstruct the original physical sampling design.

#### Detailed contingency analysis.

Pearson’s chi-square was used to measure association between ware and standardized decoration within each aggregate. Because the tables were sparse, p-values were estimated from 99,999 random tables with the same row and column totals using chisq.test(..., simulate.p.value = TRUE) in R. Association strength was summarized with Cramér’s V, calculated as


$$\begin{array}{c} V=\sqrt{\frac{\chi^{\text{2}}}{n\text{min}(r-\text{1},c-\text{1})}} \end{array}$$

where n is the table total and r and c are the numbers of rows and columns. Because the detailed classification tables differ in size, Cramér’s V was interpreted within each aggregate rather than compared directly between them [7].

Adjusted standardized residuals were used to identify cells contributing most strongly to the global association. Their p-values were corrected for multiple comparisons using the Benjamini–Hochberg procedure [20]. Interpretation emphasizes cells with expected counts of at least five; residual results remain exploratory because the cells are dependent and the tables are sparse. Correspondence-analysis coordinates, contributions, and measures of representation are reported in the supplement. Ware and decoration points are interpreted within their respective sets, and distances between the two kinds of points are not treated as direct pairwise associations [21].

#### Hellinger comparisons.

Hellinger distance was calculated as


$$\begin{array}{c} H=\sqrt{\frac{\text{1}}{\text{2}}\sum_{i}{\left(\sqrt{p_{i}}-\sqrt{q_{i}}\right)}^{\text{2}}} \end{array}$$

Six aggregate-level Hellinger estimands were calculated. Three estimands on identical shared support form the primary comparison: (1) shared-nine ware margins, obtained by summing the 9 × 6 table across technique; (2) shared-six technique margins, obtained by summing across ware; and (3) shared-support joint composition, represented by the full 9 × 6 table. Hellinger distance is calculated for each. Because these distances are not additive and joint H can reflect both marginal and association change, the difference between joint and marginal H is not interpreted as a separate effect size.

Association heterogeneity is instead tested with the homogeneous-association model described below. Three additional support-sensitivity estimands are calculated: (4) shared-ware broad joint composition, retaining the nine shared wares and all nine broad technique classes; (5) union-support broad joint composition, retaining all 11 wares and all nine broad classes; and (6) full broad-technique margins, summing all broad techniques across all wares. This design asks sequentially what is visible from wares alone, techniques alone, their joint distribution, and broader support choices.

#### Boundary-aware homogeneous-association models.

The primary 2 aggregate × 6 technique × 9 ware model includes aggregate × technique, aggregate × ware, and technique × ware terms while omitting the three-way interaction. Under this homogeneous-association null, the aggregates are allowed to differ in ware margins and technique margins, while a common ware–technique association is fitted across aggregates. Rejection therefore provides evidence of aggregate-specific ware–technique structure under the fitted sherd-count model that is not accounted for by the two marginal compositions alone. The same model is fitted after Midden is removed. A 3 unit × 6 technique × 9 ware model evaluates whether Stone Rooms, Midden, and Late Platforms share a common ware–technique association. The test does not identify elapsed time as the cause of heterogeneity. Its Poisson calibration does not incorporate clustering of sherds within vessels, because vessel identities and refits are unavailable in the matrices.

The nominal complete-table degrees of freedom are inappropriate when observed two-way margins are zero and the maximum-likelihood fit lies on the boundary. Active support was therefore defined as the cells whose three corresponding observed two-way margins were positive. The effective residual dimension was calculated as the number of active cells minus the rank of the two-way model matrix restricted to that support. Cells excluded by zero observed margins were treated as computational boundary cells rather than archaeologically impossible structural zeros.

Sparse-table calibration used 9,999 Poisson parametric-bootstrap tables generated under each fitted homogeneous-association model. Active support and model rank were recomputed for every simulated table, and a bootstrap probability was reported only when all 9,999 simulated fits converged and produced finite deviances. The bootstrap probability was calculated as pboot=(b + 1)/(B + 1), where b is the number of simulated deviances at least as large as the observed deviance and B = 9,999. The procedure is conditional on the fitted Poisson model and does not reconstruct the original physical sampling design.

Departures from homogeneous association were localized using signed Poisson deviance residuals and cellwise contributions to G^2^. These contributions are treated as dependent model diagnostics rather than independent cellwise tests. Complete fitting tolerances, iteration limits, convergence diagnostics, observed and fitted counts, residuals, cellwise contributions, and bootstrap distributions are reported in S1 File.

#### Uncertainty for Hellinger distances.

Fixed-count multinomial resampling generated 9,999 replicates using plug-in cell probabilities estimated from each observed count vector while holding the corresponding total n fixed. Unit-pair comparisons condition on the corresponding unit totals; pooled aggregate comparisons condition on the realized aggregate totals. Because aggregate matrices are cellwise sums of separately sampled analytical units, these intervals describe uncertainty around the realized pooled sherd profiles and do not reconstruct the original unit-level sampling design. A parallel Dirichlet(count + 0.5) analysis assesses sensitivity to sparse and zero cells. Neither procedure recovers unknown collection denominators, sampling fractions, or vessel-level dependence.

#### Reproducibility.

The quantitative results are retained from analysis package v12.4, which records an end-to-end R 4.5.2 execution on 19 September 2026 and validation of all three 9,999-replicate homogeneous-association bootstraps. The workflow reads five analytical-unit matrices, checks their aggregation, and generates the reported outputs; source(“R/00_run_all.R”) runs it from the package root. The revision independently checks the point Hellinger distances, observed model deviances, and archived bootstrap distributions against the manuscript. Inputs, scripts, concordance, execution records, and reference outputs are identified in the Data Availability statement.

#### Permits and ethics.

The Wacheqsa fieldwork was conducted as part of the Stanford Archaeological Project at Chavín de Huántar under authorizations issued by the Instituto Nacional de Cultura del Perú: Resolución Directoral Nacional Nº 824/INC, dated 4 August 2003; Nº 704/INC, dated 23 August 2004; and Nº 902/INC, dated 4 July 2005. All necessary permits were obtained for the study described, which complied with all relevant regulations.

The ceramic assemblage is curated at the Museo Nacional de Chavín, Ancash, Peru. The present study involved no new excavation, destructive sampling or export of archaeological materials. The article analyzes previously classified ceramic count matrices derived from curated materials; the reproducible analytical object is the finalized unit-level count matrices and associated R workflow. The author is a member of the College of Peruvian Archaeologists (COARPE) and is registered in the Peruvian Ministry of Culture’s Register of National Archaeologists (RNA).

## Results

### Primary comparison: ware, technique, and joint composition

On the identical 9-ware × 6-technique common support, the earlier–later contrast is H = 0.430 (95% fixed-count interval 0.392–0.481) for ware margins, H = 0.588 (0.551–0.634) for technique margins, and H = 0.734 (0.714–0.793) for joint ware × technique composition (Table 5). These distances answer different questions and are not additive. The association-specific test is the homogeneous-association model: after allowing aggregate-specific ware and technique margins, a common ware–technique association is rejected (G^2^ = 118.59; effective residual dimension = 28; 0 of 9,999 bootstrap deviances at least as large as observed; pboot = 0.0001). Under its assumptions, the model identifies association differences not accounted for by the aggregate-specific margins. Their chronological or depositional cause cannot be determined by this test.

**Table 5:** Marginal compositional differences, joint ware–technique divergence, and association heterogeneity between earlier and later Wacheqsa assemblages.

Comparison	n1 / n2	Ware H [95%]	Technique H [95%]	Joint H [95%]	G^2^ (eff. dim.)	pboot
Urabarroid vs Janabarroid	334 / 822	0.430 [0.392–0.481]	0.588 [0.551–0.634]	0.734 [0.714–0.793]	118.59 (28)	0.0001
Without Midden	334 / 230	0.352 [0.308–0.423]	0.560 [0.512–0.625]	0.733 [0.712–0.797]	90.16 (24)	0.0001

Note: All Hellinger estimates use the same 9-ware × 6-technique support. Ware H sums across techniques; technique H sums across wares; joint H compares the vectorized 9 × 6 table. Brackets are 95% fixed-count multinomial resampling intervals conditional on realized totals and plug-in probabilities. The three Hellinger distances are parallel effect-size summaries and are not additive. G^2^ is from the boundary-aware homogeneous-association model, which allows aggregate-specific ware and technique margins while constraining ware–technique association to be common. Both bootstrap tests used 9,999 successful refits with zero failures and zero exceedances. Dirichlet sensitivity summaries are reported in S1 File.

The leave-Midden-out analysis yields the same substantive result. Removing the numerically dominant Midden reduces ware-margin H to 0.352 (0.308–0.423) and technique-margin H to 0.560 (0.512–0.625), while joint H remains 0.733 (0.712–0.797). The corresponding homogeneous-association model also rejects a common association (G^2^ = 90.16; effective residual dimension = 24; 0 of 9,999 bootstrap exceedances; pboot = 0.0001). Thus the principal association result is not generated solely by the largest refuse sample, although removing Midden does not separate chronology from activity composition or formation process.

Early Platforms supplies 93.8% of the earlier analytical sample and Midden 72.3% of the later sample. The leave-Midden-out result reduces dependence on the largest later deposit, but the earlier comparison remains dominated by Early Platforms. Water Flood contributes only 17 shared-support observations.

### Detailed association structure and sparsity

The detailed normalized matrices also show strong within-aggregate ware–decoration structure, although part of that structure reflects the conceptual overlap between ware finish and surface treatment. Urabarroid yields χ^2^ = 698.10 on nominal df = 162, Cramér’s V = 0.469, and 0 of 99,999 simulated statistics at least as large (PMC = 0.00001). Janabarroid yields χ^2^ = 1,517.83 on nominal df = 252, V = 0.420, and 0 of 99,999 simulated statistics at least as large (PMC = 0.00001). Expected counts below five account for 90.0% of Urabarroid cells and 86.2% of Janabarroid cells; 74.2% and 49.3%, respectively, are below one. These results establish global classificatory structure but, because of sparsity and partial classificatory dependence, individual residuals are treated as exploratory. Complete statistics are reported in S1 File.

Residual diagnostics identify several ware–decoration combinations contributing strongly to the normalized within-aggregate structure, including earlier associations involving Gray Smoothed and Gray Polished wares with incision/modeling and later associations involving Black Polished and brown wares with stamping/impression. Because the detailed tables are sparse and partly classificatorily dependent, these cellwise patterns are treated as exploratory; complete observed counts, expected counts, residuals, and multiplicity-adjusted values are reported in S1 File.

### Unit composition and contextual sensitivity

Table 6 summarizes the analytical corpus and principal broad-technique percentages for the five analytical units.

**Table 6:** Analytical corpus and principal broad-technique percentages by unit.

Unit	Aggregate	n	Shared-six n	Share of aggregate analytical sample (%)	Incision (%)	Modeling (%)	Stamping/impression (%)
Early Platforms	Urabarroid	330	317	93.8	67.0	20.6	1.8
Water Flood	Urabarroid	22	17	6.3	45.5	4.5	27.3
Stone Rooms	Janabarroid	215	183	22.5	27.0	0.5	54.4
Midden	Janabarroid	691	592	72.3	21.7	2.5	66.0
Late Platforms	Janabarroid	50	47	5.2	26.0	0.0	68.0

Note: Percentages use complete nine-class unit profiles and do not sum to 100 because only three classes are shown. Aggregate share describes the analytical corpus, not archaeological prevalence.

Table 7 reports technique-margin and shared-support joint Hellinger distances among the analytical units. Aggregate and leave-Midden-out comparisons are reported in Table 5.

**Table 7:** Unit-level shared-six technique-margin and shared-support joint Hellinger distances.

Comparison	n1 / n2	Technique-margin H [95% resampling]	Joint H [95% resampling]	Role
Early Platforms-Stone Rooms	317 / 183	0.584 [0.532–0.656]	0.756 [0.734–0.821]	Earlier-later
Early Platforms-Midden	317 / 592	0.640 [0.601–0.689]	0.786 [0.766–0.844]	Earlier-later
Early Platforms-Late Platforms	317 / 47	0.649 [0.574–0.741]	0.846 [0.827–0.924]	Aggregate comparison; possible chronological overlap; small n.
Stone Rooms-Midden	183 / 592	0.133 [0.090–0.207]	0.361 [0.342–0.444]	Later contexts
Stone Rooms-Late Platforms	183 / 47	0.130 [0.084–0.232]	0.432 [0.416–0.583]	Later contexts; small later n
Midden-Late Platforms	592 / 47	0.171 [0.137–0.250]	0.484 [0.450–0.617]	Later contexts; small later n

Note: Technique-margin H compares six technique totals after summing across nine shared wares. Joint H compares the vectorized 9-ware × 6-technique composition. Brackets are fixed-count parametric resampling intervals conditional on the observed totals and plug-in probabilities. Joint H is not an association-only statistic.

Fig 4 visualizes the analytical-unit broad-technique profiles and the corresponding shared-support Hellinger distances.

**Fig 4:**
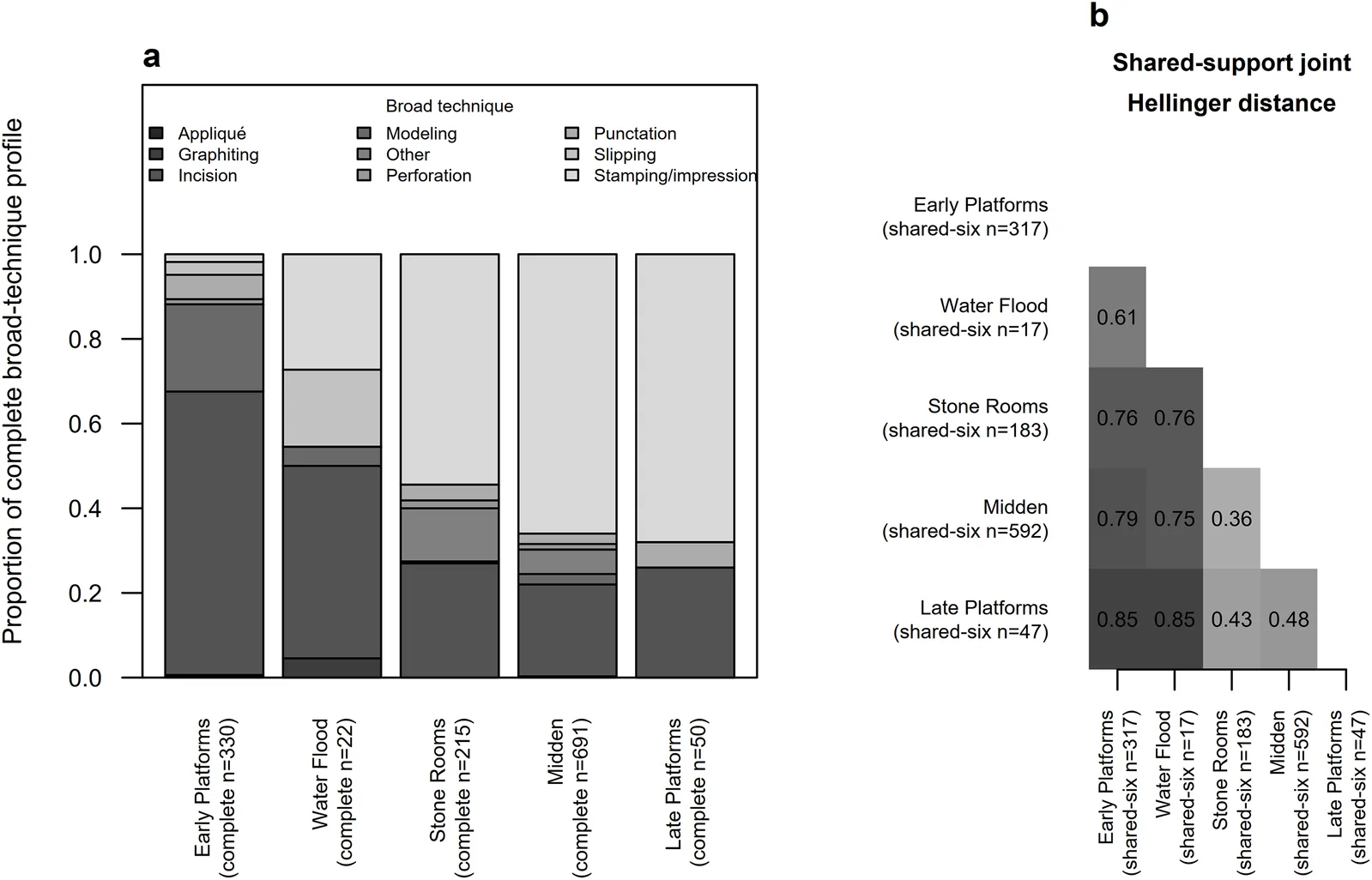
Analytical-unit ceramic composition. Panel a shows complete broad-technique proportions for the five units, with complete-profile sample totals. Panel b shows lower-triangle shared-support 9-ware × 6-technique Hellinger distances and the corresponding shared-six totals; darker shading indicates greater distance. Comparisons involving Water Flood (n = 17) and Late Platforms (n = 47) are based on small shared-support profiles. Joint H measures total common-support compositional divergence and is not an association-only statistic.

### Association heterogeneity and support sensitivity

The association-heterogeneity result in Table 5 is estimated on boundary-restricted support. Of 108 nominal cells, 84 are active after 12 zero technique × ware margins exclude 24 cells from the fitted support; the restricted model rank is 56 and the effective residual dimension is 28. Complete active-support, rank, fitted-count, convergence, and residual diagnostics are reported in S1 File.

After Midden is removed, 75 cells remain active and the effective residual dimension is 24. Among Stone Rooms, Midden, and Late Platforms, 93 cells are active and the effective residual dimension is 34; the later-unit model also rejects homogeneous ware–technique association (G^2^ = 80.63; 0 of 9,999 bootstrap exceedances; pboot = 0.0001). Thus later contexts with broadly similar technique margins still differ in how techniques are distributed among wares. These models identify association heterogeneity under the sherd-count model but do not determine its archaeological cause.

Cellwise decomposition shows that the aggregate and later-unit departures are distributed across multiple ware–technique combinations involving incision, modeling, punctation, and stamping/impression rather than being driven by a single dominant cell (S1 File). Several influential cells have small fitted counts, so the decomposition is used to localize model departure rather than to make independent cell-level claims. Complete signed residuals, contributions to G^2^, percentage shares, and ranks are reported in S1 File.

Conditional uncertainty and zero-cell sensitivity do not overturn the primary result. On shared support, ware-margin H is 0.430 (95% fixed-count interval 0.392–0.481; Dirichlet median 0.431, 95% sensitivity 0.388–0.473), technique-margin H is 0.588 (0.551–0.634; Dirichlet median 0.588, 0.545–0.627), and joint H is 0.734 (0.714–0.793; Dirichlet median 0.698, 0.661–0.732). Without Midden, the corresponding values are ware H = 0.352 (0.308–0.423; Dirichlet median 0.357, 0.301–0.413), technique H = 0.560 (0.512–0.625; Dirichlet median 0.556, 0.501–0.608), and joint H = 0.733 (0.712–0.797; Dirichlet median 0.672, 0.623–0.714). Retaining all nine broad classes among the nine shared wares gives joint H = 0.752 (0.733–0.807), and retaining the full 11-ware union with all nine broad classes gives H = 0.763 (0.745–0.815). Full estimands and sensitivity ranges are reported in S1 File. Thus retaining the aggregate-specific categories raises the point estimate of joint H from 0.734 to 0.763, a difference of approximately 0.030. These estimates use different supports and renormalized profiles; their difference is not a formal decomposition of the contribution of excluded observations. Both support choices retain a substantial descriptive contrast.

### Ware-specific technique-profile differences

Among wares with at least ten shared-support observations in each aggregate, H is highest for CGP (H = 0.833; 95% fixed-count resampling interval 0.768–0.957), followed by CBS (0.695; 0.615–0.795), CGS (0.689; 0.627–0.756), CBP (0.668; 0.587–0.810), and CBrP (0.612; 0.496–0.822). Dirichlet(count + 0.5) sensitivity medians are 0.785, 0.602, 0.623, 0.650, and 0.605, respectively, and retain the same five largest differences among wares meeting the reporting threshold. COP (9/8), CRS (1/30), and CBrS (7/89) remain low-information comparisons; COS (19/25) meets the reporting threshold but has a smaller distance (H = 0.258; 0.086–0.432) (Table 8; Fig 5).

**Table 8:** Ware-specific shared-six Hellinger distances and conditional uncertainty.

Ware	Urabarroid / Janabarroid n	Shared-support H	95% fixed-count resampling	Dirichlet median [95% sensitivity]	Information
CGP	66 / 57	0.833	0.768–0.957	0.785 [0.677–0.865]	meets reporting threshold
CBS	26 / 56	0.695	0.615–0.795	0.602 [0.464–0.711]	meets reporting threshold
CGS	136 / 48	0.689	0.627–0.756	0.623 [0.519–0.704]	meets reporting threshold
CBP	45 / 402	0.668	0.587–0.810	0.650 [0.551–0.731]	meets reporting threshold
CBrP	25 / 107	0.612	0.496–0.822	0.605 [0.463–0.719]	meets reporting threshold
COP	9 / 8	0.508	0.259–0.858	0.452 [0.219–0.664]	Low information
CRS	1 / 30	0.353	0.227–0.475	0.465 [0.188–0.749]	Low information
CBrS	7 / 89	0.293	0.187–0.636	0.363 [0.164–0.563]	Low information
COS	19 / 25	0.258	0.086–0.432	0.231 [0.106–0.377]	meets reporting threshold

Note: Fixed-count intervals condition on observed zeros. Dirichlet(count + 0.5) ranges regularize zero cells and describe probability-profile sensitivity, not vessel-level uncertainty. Rows with either total below 10 are flagged as low information.

**Fig 5:**
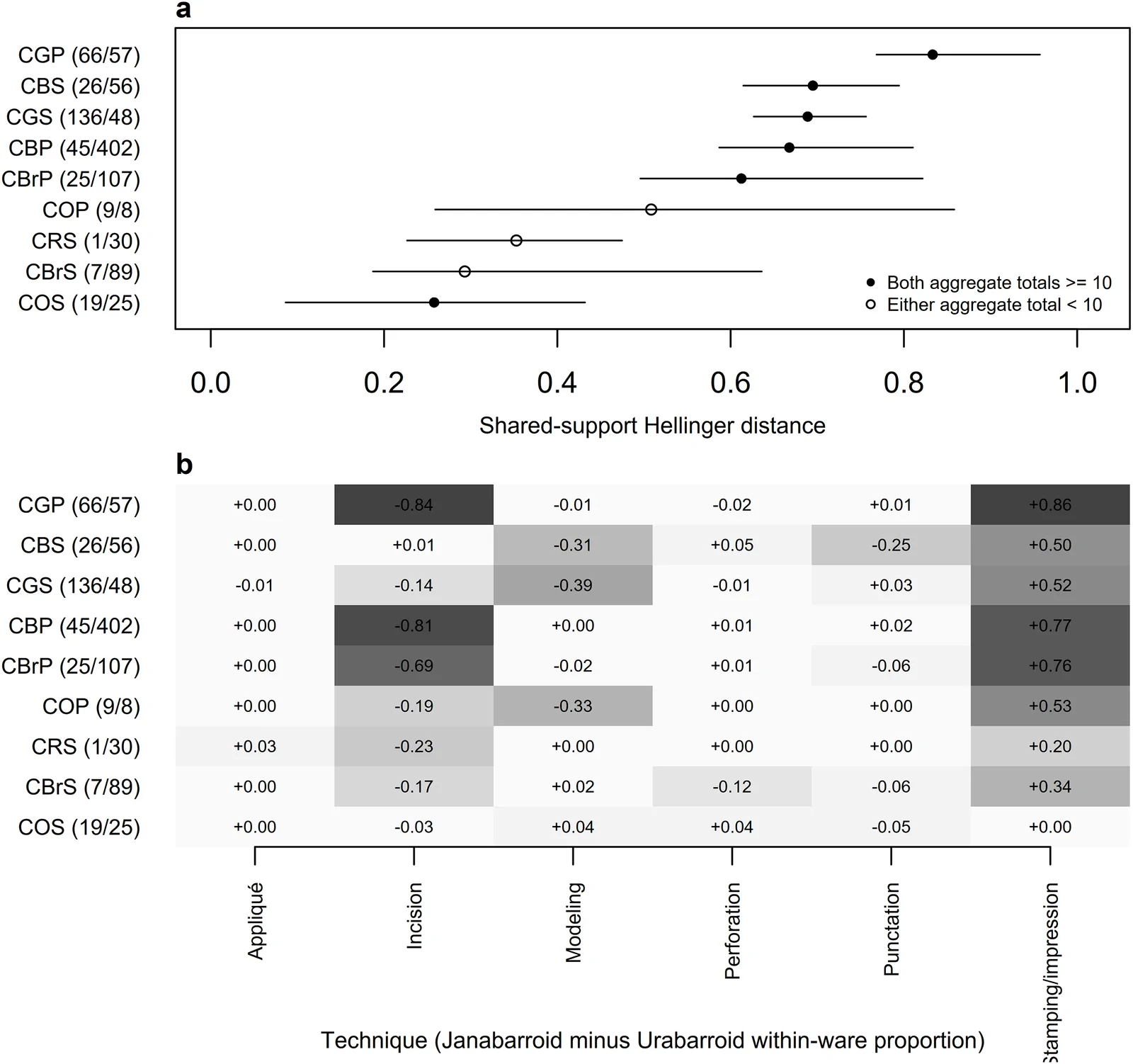
Ware-specific shared-six technique-profile differences between the Urabarroid and Janabarroid aggregates. Panel a reports shared-support Hellinger distance with 95% fixed-count parametric resampling intervals. Filled symbols indicate at least ten observations in each aggregate; open symbols indicate that either aggregate has fewer than ten. Panel b reports Janabarroid minus Urabarroid within-ware proportional change for the six shared techniques. Shading represents absolute change; signed values show direction.

Within shared-six profiles, incision falls by 0.841 in CGP, 0.809 in CBP, and 0.688 in CBrP, while stamping/impression rises by 0.860, 0.767, and 0.761. In CGS, modeling falls by 0.390 and stamping/impression rises by 0.521. CBS differs mainly through lower modeling and punctation and a 0.500 increase in stamping/impression. These are within-ware proportional changes, not changes in overall ware abundance.

## Discussion

### Continuing wares and differing decorative associations

Nine of the eleven recorded ware categories occur in both aggregates. This continuity concerns macroscopic ware categories, not continuity of individual vessels, potters, production systems, or social groups. Within the common-support profiles, Gray Polished incision falls from 89.4% to 5.3%, while stamping/impression rises from 0% to 86.0%. In Black Polished, incision falls from 93.3% to 12.4% and stamping/impression rises from 4.4% to 81.1%; in Brown Polished the corresponding values are 80.0% to 11.2% and 8.0% to 84.1% (Fig 5; Table 8). These are conditional percentages among retained observations within each ware, rather than the abundance of those wares in the complete assemblage. They make the material contrast explicit: several continuing polished categories are associated predominantly with incision in the earlier sample and with stamping/impression in the later sample.

The pattern varies among wares. Gray Smoothed shows a pronounced reduction in modeling, whereas Orange Smoothed differs comparatively little in incision or stamping/impression. Wares with few observations provide less secure comparisons. The contrast concerns particular ware–technique combinations rather than a uniform replacement of techniques.

The homogeneous-association model shows that the aggregate-specific ware and technique margins alone do not account for the observed pairings (Table 5). The archaeological interpretation rests on these combinations within their depositional contexts.

### Context comparisons and the influence of midden

Direct comparisons show that Early Platforms differs substantially from each later context in both technique frequencies and joint composition (Tables 6 and 7). The contrast is therefore visible without pooling the later samples. These comparisons do not isolate a temporal mechanism, and the Late Platforms estimate requires particular caution because its retained sample contains only 47 observations.

Stamping/impression occurs in 6 of 330 Early Platforms observations (1.8%), compared with 117 of 215 in Stone Rooms (54.4%), 456 of 691 in Midden (66.0%), and 34 of 50 in Late Platforms (68.0%). Its prominence recurs across three later depositional settings rather than being confined to Midden.

The contrast persists after removing Midden (Table 5), so the largest later sample does not account for the aggregate result. Activity and formation history may still contribute to the differences.

Stone Rooms, Midden, and Late Platforms have broadly similar technique frequencies but distinct joint profiles (Table 7); their common-association model is also rejected (S1 File). The shared prominence of stamping/impression thus coexists with context-specific combinations, and the pooled Janabarroid profile should not be treated as a homogeneous ceramic package.

### Depositional histories and ceramic representation

The deposits select and accumulate ceramics through different processes. Early Platforms combines floors, platform surfaces, and low-density fills; Stone Rooms includes occupied spaces, cleaning episodes, and subsequent fills. Differences between these locally superposed units may therefore reflect use and deposition as well as elapsed time.

Midden contains repeated refuse deposits; its cumulative assemblage need not represent a single event or a single vessel-use setting [9,22]. Late Platforms comprises construction and closure-related fills, in which stamped material may have been incorporated through redeposition. Its presence does not by itself demonstrate contemporary use or a termination ritual.

Water Flood’s stamping/impression frequency of 27.3% represents six observations out of 22. Selective transport, redeposition, and mixing could contribute to this water-laid profile. The sample is too small, and its formation too distinct, to establish a transitional ceramic stage.

### Ceramic repertoires and the organization of social practices

The differing combinations of continuing wares and decorative techniques are consistent with differences in the ceramic repertoires represented in activity settings and their associated deposits. The archaeological question is how those differences relate to the organization of activities and the processes through which ceramic material entered the deposits.

The spatial and depositional evidence gives these differences a social setting. Early Platforms has been interpreted as evidence of domestic and provisioning activities, whereas Stone Rooms represents segmented architectural spaces and Midden is associated with recurrent collective consumption [9,10,22]. The contrast between incision-dominated Early Platforms and stamping-rich Stone Rooms and Midden suggests that different decorated repertoires accompanied differences in activity organization and in the ceramic material entering those settings. Vessel use, access, circulation, and production are possible contributors, alongside depositional filtering, preservation, and fragmentation.

The wider contextual evidence for cooking and serving vessels, animal remains, and associated materials supports recurrent collective consumption in Midden [9,22]. The abundance of stamping/impression in its decorated sherd sample raises the question of how decorated vessels participated in these practices. If used in serving or presentation, decorated surfaces could have contributed to the visual setting of commensality. The present matrices do not cross-classify decoration with vessel form or use evidence, so they cannot establish that role or demonstrate that decoration communicated rank, affiliation, or ritual authority. The contextual association nevertheless provides a basis for examining the relationship between ceramic appearance, consumption, and social interaction.

The distribution of stamping/impression across Stone Rooms, Midden, and Late Platforms also directs attention to the movement of ceramics between activity areas and deposits. Cleaning, transfer, and redeposition could connect refuse accumulation with the incorporation of decorated material into construction and closure fills. These contexts therefore need not represent independent populations of vessels in contemporary use. Their different joint profiles further indicate that the shared prominence of stamping/impression encompassed context-specific ware combinations. A broad decorative repertoire could be represented differently through activities, disposal, and subsequent reworking within the sector.

### Stratigraphic order and chronological interpretation

Superposition supports local earlier–later comparisons: Stone Rooms overlies Early Platforms in several exposures, and Midden overlies or cuts Water Flood. Correlations across partly disconnected networks additionally rely on spatial, architectural, depositional, and chronological evidence [9,10,12,22]. The affinity labels supply comparative terminology rather than independent calendar ages.

Local superposition supports a temporal component in the relationship between ceramic differentiation and the reorganization of activity settings. At Wacheqsa, Stone Rooms above Early Platforms provides a basis for considering how decorated repertoires differed as the use and partitioning of space changed. The published aggregate chronology supplies a broader temporal framework but does not directly date the transition in individual ceramic practices. Architectural reorganization in the monumental core [11,13–15] makes the relationship between local practices and wider changes at Chavín a relevant historical question. The present evidence supports examining that relationship; demonstrating synchronicity or a shared institutional mechanism requires explicit comparison of independently constrained model estimates and their uncertainty across sectors.

The results therefore describe a local, stratigraphically structured contrast. They neither establish a uniform site-wide ceramic trajectory nor independently validate the boundaries of Burger’s phases. The absence of a separately coded Chakinani sample in these matrices does not establish its archaeological absence. Chakinani should nevertheless be treated as a proposed chronological subdivision whose site-wide validity requires demonstration.

### Coding uncertainty and limits of inference

The association analysis describes the single labels preserved in the original records. If sherds carried multiple treatments, the priority given to one treatment could influence ware–technique frequencies. Even a consistently applied priority rule could affect comparisons if treatment co-occurrence differed among wares or contexts. Because additional treatments and the original selection rule are not preserved, this effect cannot be quantified. The observed contrast therefore concerns recorded classifications rather than the complete distribution of treatments on individual sherds.

Common-support selection also affects representation. Of 352 earlier observations, 18 (5.1%) are excluded; of 956 later observations, 134 (14.0%) are excluded. Table 4 separates ware-only, technique-only, and overlapping exclusions. Retaining all categories slightly increases the measured joint contrast (S1 File). Because the profiles are renormalized, this increase cannot be interpreted as an additive contribution from particular excluded categories.

Unknown vessel clustering limits the precision of inference: the reported intervals and bootstrap probabilities apply to the sherd-count model and do not quantify uncertainty among vessels.

The observed combinations constrain possible explanations, but the matrices cannot distinguish changes in production or circulation from differences in vessel use and deposition. Refits could identify fragments from the same vessel; technological and compositional analyses could assess production and circulation; use-related evidence and closer examination of individual deposits could clarify function and redeposition [23,24].

## Conclusions

Continuing ware categories occur in markedly different decorative combinations across the sampled Wacheqsa contexts. These differences extend beyond changes in ware and technique frequencies under the fitted sherd-count model, and persist in direct context comparisons and after removing the largest later deposit, Midden.

Archaeologically, the contrast is consistent with a reconfiguration of decorated ceramic repertoires across settings associated with domestic and provisioning activities, segmented architectural spaces, collective consumption, and construction or closure. The persistence of macroscopic ware categories did not entail stable decorative associations or equivalent representation across those settings. The findings therefore connect ceramic differentiation to the organization of activities and the processes through which vessels entered deposits. Local superpositions support a temporal component, while use, circulation, and redeposition remain possible, potentially interacting explanations. The broader anthropological implication is that continuity in familiar material categories can coexist with changes in their participation in social practices; the present evidence identifies that possibility without independently establishing institutional transformation.

## Supporting information

S1 File Supplementary information archive. Compressed archive containing S1–S3 Figs and S1–S5 Tables: the Wacheqsa context-level Harris matrix; correspondence analyses; lexical concordance and broad-technique assignments; normalized aggregate and unit matrices; Hellinger comparisons and uncertainty analyses; and boundary-aware homogeneous-association model diagnostics and bootstrap distributions.
(ZIP)
