# Supplementary material for: Changes in ware–decoration associations in ceramic assemblages from Wacheqsa, Chavín de Huántar, Peru
Source: PLoS One. 2026 Oct 29;21(10):e0361889. doi: 10.1371/journal.pone.0361889 (PMC13718927; doi:10.1371/journal.pone.0361889)
Supplement: Supplementary file 1 [file pone.0361889.s002.pdf]

Review: PONE-D-26-35517

I recommend major revision.

This is a careful quantitative reanalysis of a legacy ceramic corpus from Wacheqsa, Chavin de Huantar. The paper is strongest when it describes differences in the organization of ware and decoration across stratigraphically differentiated contexts, while being honest about the limits of the data. The author is also clear that these are sherd counts rather than reconstructed vessels and that chronology, activity, formation processes, and fragmentation remain confounded.

My main concern is with the paper's contrast between decoration and ceramic types. The paper does show that decoration changes within continuing ware categories. It also shows that ware-technique associations differ after the separate ware and technique margins are taken into account. What it does not yet show is that looking at decoration produces more, or different, archaeological interpretation than looking at types or wares alone. This is a reasonable assertion, but I did not find the paper firmly established this.

The introduction says that ceramic sequences often emphasize the appearance, disappearance, and frequency of types, while this paper identifies a different form of change: the redistribution of techniques among continuing wares. That is a sensible and potentially useful point. The current analysis does test for and describe that reorganization within the classified corpus.

The stronger implication is still unresolved. The paper never defines clearly what it means by a ceramic "type" in relation to a ware and a decoration. This is important because archaeological types may already include decoration, while the ware labels in this paper partly include surface finish. It is therefore hard to know what, exactly, the paper is adding to a type-based analysis.

I would ask the author to make the comparison directly. Using the same count matrices, show a ware/type-only analysis, a decoration-only analysis, and the joint ware-by-decoration analysis. Then say what conclusion changes when decoration is included. The existing framework should allow this without new fieldwork or new data. The joint results alone do not settle the issue, since they combine differences in ware frequency, technique frequency, and the association between them.

If the joint analysis shows a result that is not visible in the separate analyses, that should become the paper's central contribution. If it does not, the paper should be framed more modestly as a quantitative description of ceramic compositional reorganization. Either outcome would be useful, but the present wording leaves the question open.

The Wacheqsa sequence is at once too detailed and not detailed enough. I wanted a clearer high-level orientation before the excavation details. At line 87, for example, Wacheqsa is described as immediately north of "the monumental core," but the reader should be told that this means the monumental core of Chavin de Huantar.

The opening context section should first explain where Wacheqsa is, that the paper compares five stratigraphically differentiated depositional contexts, how those contexts are grouped into earlier and later aggregates, and why their formation histories matter for interpreting ceramic composition. It should also say early that this is neither a five-stage sequence nor a population estimate.

Urabarroid and Janabarroid need to be defined before their first use in the discussion of context assignment. I initially read them as phases, then as ceramic types, before the manuscript made clear that they are local context aggregates identified by ceramic affinity. A short definition at first use would solve this problem.

Much of the excavation volume, artifact-density, and inventory information can move to Table 1 or the supplement. The prose should keep only what is needed for the comparison: relative

stratigraphic position, the water-laid character of Water Flood, the cumulative-refuse character of Midden, and the closure/fill character of Late Platforms. The landslide needs only a brief mention if it explains reliance on excavated stratigraphy. The microbotanical results seem out of place unless they are part of the analysis.

I would also add a compact table in the main text listing the nine shared ware codes and full names, along with the six broad decorative techniques used in the principal comparison. The full concordance can remain in the supplement. At present, the reader has to assemble the analytical vocabulary from several paragraphs and external files.

I am less convinced that the evolutionary framework needs so much space. The author is clear that the paper does not test learning, drift, selection, or production organization. In that case, the evolutionary material should be shorter and kept as a statement about questions for future work. Otherwise, the Introduction needs explicit evolutionary predictions that these data can evaluate.

I want to congratulate the author on the reproducibility materials. I was able to run the documented workflow from the supplied count matrices, generate the expected outputs, and pass the project's validation checks. The fixed seeds, lineage checks, session information, reference results, generated figures, and checksum manifest are all excellent. They make the numerical analysis much easier to assess and trust.

The OSF project would benefit from a better public front page. It currently has only a one-sentence description, no wiki, no visible license, and no immutable registration. The data and supplementary materials should have a CC BY 4.0-compatible license, and the R code should have a clear open-source license such as MIT or BSD.

The OSF overview should also identify the manuscript version, explain the difference between the OSF title and the submitted title, map `R.zip`, the supplementary tables, and the figures to the paper, and give the one-command reproduction route.

The paper has a strong foundation, especially in its reproducible analysis. Major revision is needed to clarify and, if warranted, demonstrate the claimed value of decoration beyond types or wares, and to make the contextual and conceptual framing easier to follow.
