# Supplementary material for: Changes in ware–decoration associations in ceramic assemblages from Wacheqsa, Chavín de Huántar, Peru
Source: PLoS One. 2026 Oct 29;21(10):e0361889. doi: 10.1371/journal.pone.0361889 (PMC13718927; doi:10.1371/journal.pone.0361889)
Supplement: Supplementary file 2 [file pone.0361889.s004.docx]

**Response to the Academic Editor and Reviewers**

Manuscript PONE-D-26-35517R1
Ceramic change at Wacheqsa, Chavín de Huántar: Changing associations between ware and decorative technique in an Andean Formative assemblage

Dear Dr. Mroczek and Reviewers,

Thank you for the time invested in evaluating the manuscript and for giving me the opportunity to revise it. I took the concerns raised in the decision letter and the two reports as requiring changes to the argument, not simply additions to the text.

The most substantial revision concerns the purpose of the joint ware–decoration analysis. The paper now compares ware composition, technique composition, and joint ware × technique composition on exactly the same analytical support, and then tests separately whether ware–technique association changes after differences in the two margins have been fitted. I have also rewritten the terminology and the treatment of the inherited ceramic sequence, expanded the archaeological presentation of Wacheqsa and the five analytical units, made the sample structure and inferential limits explicit throughout, shortened the evolutionary discussion, and rebuilt the Supporting Information and reproducibility package around the final unit-derived analysis.

The revised manuscript is cited below by page and numbered manuscript lines.

**Academic Editor — Dr. Przemysław Mroczek**

Comment 1

“The main issue is the contribution of the joint ware-decoration analysis. The revised manuscript should compare, using the existing dataset, ware-only composition, decoration-only composition and joint ware × decoration structure. The author should then state clearly what is learned from the joint analysis that is not already apparent from the two marginal patterns.”

Response

I agree. This became the central analytical revision.

The earlier and later assemblages are now compared on identical support comprising nine wares and six broad techniques. Three Hellinger comparisons are reported separately:

ware composition, H = 0.430 (95% fixed-count interval 0.392–0.481); technique composition, H = 0.588 (0.551–0.634); and joint ware × technique composition, H = 0.734 (0.714–0.793).

I also make explicit that these three distances are not additive. In particular, the larger joint H is not interpreted as an association effect. Joint H can incorporate both marginal change and association change.

For that reason, I added a boundary-aware homogeneous-association log-linear model. The model allows the two aggregates to have different ware margins and different technique margins while constraining their ware–technique association to be common. That model is rejected (G² = 118.59; effective residual dimension = 28; pboot = 0.0001). All 9,999 parametric-bootstrap refits converged, with no simulated deviance reaching the observed value.

The added information from the joint analysis is therefore now stated precisely: the later assemblage differs not only in the abundance of wares and techniques, but also in the way decorative techniques are distributed among the continuing ware categories.

This distinction now structures the Introduction, Methods, Results, Discussion, and Conclusions.

Location in revised manuscript: pp. 3–4, lines 42–99; pp. 13–14, lines 301–349; pp. 15–16, lines 350–399; p. 21, lines 488–513; pp. 25–26, lines 592–643. The final Methods explicitly distinguishes the six Hellinger estimands and states why association heterogeneity is tested separately. The principal Results report the three marginal/joint distances and the association-specific model.

Comment 2

“The terminology also needs revision. ‘Type’, ‘ware’, ‘decoration’, ‘technique’, Urabarroid and Janabarroid should be defined clearly and used consistently.”

Response

I revised the terminology throughout the paper.

In the revised manuscript, ware denotes the macroscopic color-plus-surface-finish categories recorded in the surviving matrices. Detailed decoration denotes the standardized decorative or surface-treatment label assigned to a sherd. Broad technique is the higher-order analytical grouping used in the comparative analysis. Type is reserved for historically published composite typological constructs such as Urabarriu, Chakinani, and Janabarriu.

Urabarroid and Janabarroid are defined at first use as local Wacheqsa aggregate labels. Urabarroid denotes Early Platforms + Water Flood; Janabarroid denotes Stone Rooms + Midden + Late Platforms. The *-oid* suffix indicates ceramic affinity and does not designate an independently defined phase or ceramic type.

I also clarify that ‘ware’ in this dataset is an operational macroscopic color-plus-surface-finish category. I do not use it as a synonym for technological style or as a direct measure of manufacturing technology, paste recipe, or production organization

The same terminology is used consistently in the Methods, Results, tables, figures, and Discussion.

Location in revised manuscript: p. 3, lines 42–72; pp. 6–7, lines 130–182; pp. 10–11, lines 224–275. The operational definitions are stated explicitly in the Methods.

Comment 3

“The manuscript should distinguish the stratigraphic and contextual basis of the five analytical units from the ceramic affinities expressed by the Urabarroid and Janabarroid labels. This distinction is necessary to address the concern about circular reasoning and to avoid giving the impression that the analysis independently validates the traditional Urabarriu–Chakinani–Janabarriu sequence.”

Response

I agree that the earlier manuscript did not make this distinction clearly enough.

The revised paper now separates the archaeological context sets from the ceramic-affinity labels attached to them.

The five analytical units were constructed during the original post-excavation analysis from stratigraphic position, spatial continuity, sediment matrix, deposit geometry, architectural association, and formation history. Their principal stratigraphic relationships therefore predate the standardized ware–decoration matrices analyzed in this paper.

Ceramic affinity subsequently motivated the descriptive labels Urabarroid and Janabarroid, but those labels did not create the five context sets or their principal stratigraphic ordering.

I also explain why Burger’s types were not reconstructed as another analytical variable. Those historical types combine attributes that include finish, form, and decoration. Reconstructing a Burger-type assignment from the same attributes subsequently entered into the analysis would not provide an independent comparator. The revised paper therefore uses the recorded ware margin as the non-decoration compositional baseline.

The manuscript no longer presents its results as an independent validation of Burger’s phase sequence.

Location in revised manuscript: pp. 3–4, lines 42–99; pp. 5–8, lines 100–206; pp. 24–25, lines 566–618. The construction of the units and their relationship to the -oid labels are now described directly.

Comment 4

“The limits of the dataset also need to remain visible in the interpretation. The earlier aggregate is dominated by Early Platforms and the later aggregate by Midden. The leave-Midden-out test shows that the main numerical pattern is not caused by that sample alone, but it does not separate chronology from activity, depositional context or formation processes.”

Response

This limitation is now stated throughout the manuscript rather than only in the Discussion.

Early Platforms contributes 330 of 352 observations (93.8%) to the earlier analytical sample. Midden contributes 691 of 956 observations (72.3%) to the later sample. I therefore do not treat the aggregate contrast as balanced replication of two chronological populations.

The leave-Midden-out comparison is now reported alongside the principal aggregate result. After Midden is removed, ware H decreases from 0.430 to 0.352, technique H from 0.588 to 0.560, while joint H remains 0.733. The homogeneous-association model continues to reject a common association (G² = 90.16; effective residual dimension = 24; pboot = 0.0001).

The interpretation has been deliberately restricted. The sensitivity analysis shows that the principal association result is not produced solely by Midden. It does not remove confounding among chronology, activity composition, formation process, fragmentation, preservation, or recovery. Water Flood is also too small to provide a genuinely independent earlier replicate.

Location in revised manuscript: pp. 9–12, lines 207–300; pp. 15–16, lines 350–399; pp. 21 and 25–26, lines 488–513 and 592–643. The scope-of-inference section now states these restrictions before the inferential analyses are presented. The Results retain the same qualification after the leave-Midden-out test.

Comment 5

“Statements about changes through time should therefore be made cautiously and should refer to the analysed sherd assemblages rather than to vessel populations or ceramic production more generally.”

Response

I revised the language accordingly.

The observational unit is now stated explicitly as one classified sherd. The manuscript notes that systematic vessel refitting was not undertaken or is not retained in the analytical record, so the observations cannot be treated as independent vessel counts.

Fixed-count resampling and Dirichlet sensitivity ranges are therefore described as uncertainty or sensitivity of the analyzed sherd profiles, not of vessel populations. The conclusions are likewise restricted to stratigraphically ordered Wacheqsa sherd assemblages.

Production, selection, circulation, use, breakage, discard, and depositional filtering remain possible contributors to the observed profiles, but no one of these is inferred directly from the matrices.

Location in revised manuscript: pp. 4 and 9–14, especially lines 73–99 and 207–349; pp. 24–26, lines 566–643.

Comment 6

“The archaeological context should be presented more directly. Wacheqsa, the five analytical units and their grouping into earlier and later assemblages should be explained before the detailed analytical discussion. Urabarroid and Janabarroid should be defined at first use.”

Response

I reorganized the opening of the paper around this request.

The Introduction now begins by locating Wacheqsa immediately north of the monumental core of Chavín de Huántar, names the five analytical contexts, and defines the Urabarroid and Janabarroid aggregates before introducing the statistical problem.

The archaeological section was also expanded. The revised manuscript distinguishes Early Platforms, Water Flood, Stone Rooms, Midden, and Late Platforms by stratigraphic position, formation process, excavation context, and material inventory.

A new schematic Figure 1 provides the site and excavation orientation, while Figure 2 shows the documented exposures associated with the analytical units. Table 1 summarizes the archaeological evidence relevant to each context.

Location in revised manuscript: pp. 3–9, lines 42–223.

Comment 7

“The evolutionary discussion can also be shortened unless the author can link it to predictions that are actually tested by the present data.”

Response

I shortened and repositioned that discussion.

The revised paper does not infer conformity, prestige bias, neutral drift, selection, centralized production, or particular learning pathways from the observed frequency distributions.

Where evolutionary concepts remain, they are used only to identify hypotheses that would require additional evidence. The Discussion now specifies the kinds of evidence needed to distinguish such mechanisms, including vessel-equivalent observations and refits, technological and toolmark evidence, compositional provenance, use-wear or residue data, and finer event-level formation histories.

The paper’s contribution is therefore framed as an archaeological analysis of assemblage composition and association structure, not as a test of a specific evolutionary mechanism.

Location in revised manuscript: pp. 23–25, lines 540–618, particularly the section “What the data do not establish.”

Comment 8 — Clarity and presentation
“The writing needs significant reworking for clarity…”

Response
I revised the manuscript throughout for clarity and reduced several sources of ambiguity identified in the review. I removed the earlier formulation “continuity with redistribution,” defined the analytical terminology at first use, reorganized the archaeological context before the quantitative analysis, shortened the excavation and evolutionary discussions, and separated empirical results from possible behavioral explanations. The revised manuscript also distinguishes consistently among ware composition, technique composition, joint composition, and ware–technique association.

**Reviewer 1**

Reviewer 1’s report was written as a continuous critique rather than as numbered comments. I respond below to its main substantive points using the reviewer’s own wording.

Comment 1

“It is not clear to me what the goal of the MS is. Is it a methodological contribution? Or one that hopes to contribute to Chavin studies and the anthropological meaning of changes in pottery making practices…? Where are the humans?”

Response

This criticism led me to reorganize the paper.

The revised manuscript is not presented primarily as a demonstration of statistical methods. It asks an archaeological question: what actually changed in the Wacheqsa ceramic assemblages, and can that change be reduced to differences in ware abundance or decoration frequency alone?

The Discussion now begins with “What changed archaeologically at Wacheqsa?” The analysis shows categorical persistence in much of the ware repertoire together with changes in ware abundance, decorative emphasis, and ware–technique association.

I also expanded the treatment of the contexts in which those assemblages formed. Early Platforms are discussed in relation to localized domestic/provisioning activity; Stone Rooms in relation to segmented architectural space and a broader material inventory; Midden in relation to recurrent collective consumption, cleaning, and secondary disposal; and Late Platforms as closure/reorganization fills.

At the same time, I have avoided assigning the observed profiles directly to particular groups of people or institutions where the data do not support that inference.

Location in revised manuscript: pp. 20–24, lines 465–591.

Comment 2

The reviewer questioned the earlier formulation of “continuity with redistribution” and asked what “redistribution” meant archaeologically.

Response

I removed that formulation.

The revised manuscript now separates several different empirical observations instead of combining them under “redistribution”.

First, nine of the eleven ware categories represented across the two aggregates occur in both. That is categorical persistence.

Second, the relative abundance of those wares changes.

Third, decorative-technique frequencies change substantially, particularly through the increase in stamping/impression and decline in incision/modeling.

Fourth, the association linking techniques to continuing wares also changes.

The resulting interpretation is now phrased as substantial change within a largely persistent repertoire, or more specifically as categorical persistence accompanied by compositional and relational change.

Location in revised manuscript: Abstract, p. 2; Discussion, pp. 20–21, lines 465–513; Conclusions, pp. 25–26, lines 592–643.

Comment 3

“To me, the analysis presented amounts to an exercise in circular reasoning…”

Response

I revised the manuscript specifically to separate the contexts being compared from the historical typological labels.

The Wacheqsa analytical units were established in the original post-excavation work from stratigraphy, deposit geometry, sediment matrix, architectural association, spatial continuity, and formation history. Their principal stratigraphic ordering does not derive from the standardized ware–decoration frequency analysis reported here.

Urabarroid and Janabarroid are retained only as descriptive affinity labels attached to those context aggregates.

The paper also no longer treats Burger’s historical types as independent variables. Reconstructing those types from ware, finish, form, and decoration and then testing them using overlapping attributes would itself create the circularity raised by the reviewer.

The empirical comparison instead uses recorded ware categories, broad techniques, and their joint distribution.

Location in revised manuscript: pp. 3–8, lines 42–206; pp. 10–11, lines 224–275; pp. 24–25, lines 566–618.

Comment 4

The reviewer argued that demonstrating only that Urabarriu and Janabarriu share some attributes would have little archaeological value because this is already implicit in typological classification.

Response

I agree with that criticism, and the revised manuscript no longer treats shared categories by themselves as the principal result.

The direct marginal comparisons show what would be learned from wares alone and techniques alone. The additional association model then asks a different question: whether the same decorative techniques occur on the same wares in the same proportions once both marginal distributions are allowed to differ.

The rejection of homogeneous association demonstrates that the change is relational as well as compositional. Familiar ware categories persist, but their relationships with decorative techniques differ between the earlier and later context sets.

That is the specific information not supplied by simply observing that the named assemblages contain both similarities and differences.

Location in revised manuscript: pp. 13–16, lines 301–399; Discussion, p. 21, lines 488–513.

Comment 5

“If so, why not present distribution maps, stratigraphic sections and more data on the archaeological contexts themselves?”

Response

I added considerably more spatial and contextual information.

Figure 1 now provides a schematic site and excavation map locating Wacheqsa relative to the monumental core and neighboring sectors. Figure 2 maps the documented excavation exposures in which the analytical units occur, including the WQ-7 systematic-sampling frame.

The text now explains that apparent spatial overlap among analytical units reflects superposition in three-dimensional stratigraphy rather than mutually exclusive surface zones. The original context-level Harris matrix is also provided as S1 Fig.

Table 1 was expanded to summarize formation and stratigraphy, excavated inventory, and contextual interpretation for each analytical unit.

Location in revised manuscript: pp. 3 and 5–9, lines 42–72 and 100–223; Supporting Information caption on p. 30.

Comment 6

“What seems of value might be to try to understand how and why ceramic technologies and decorative tastes change over time…”

Response

The revised Discussion addresses the archaeological implications of the observed changes but stops short of attributing them to causes the present data cannot discriminate.

The assemblages show a marked increase in stamping/impression across all three later contexts, but the later contexts differ in their ware–technique configurations. This indicates a shared broad decorative orientation without a single homogeneous Janabarroid profile.

Possible explanations include production choices, visual or tactile preferences, vessel selection, circulation, use setting, and depositional selection. The present sherd-count matrices cannot distinguish among these alternatives.

The manuscript therefore identifies the archaeological pattern first and defines the additional evidence needed for causal interpretation rather than selecting a mechanism from the frequency data alone.

Location in revised manuscript: pp. 22–24, lines 514–591.

Comment 7

The reviewer recommended either presenting enough contextual and ceramic evidence to contribute directly to Chavín studies or reframing the paper explicitly as a methodological contribution.

Response

I chose the first route.

The revised manuscript is framed as an archaeological contribution centered on Wacheqsa and Chavín rather than as a methods paper. Statistical procedures are used to answer the archaeological question and are not presented as the primary contribution in themselves.

The Discussion now considers the ceramic change against the independently documented architectural reorganization of the monumental core. The modeled Wacheqsa transition at approximately 878–806 cal BCE broadly overlaps the onset of Black and White Stage reorganization, but the paper explicitly states that this correspondence does not establish event-level synchrony or common causation.

This is presented as a site-wide hypothesis requiring independently dated assemblages from other sectors, not as a causal conclusion.

Location in revised manuscript: pp. 23–24, lines 540–591; Conclusions, pp. 25–26, lines 592–643.

**Reviewer 2**

**Comment 1**

“My main concern is with the paper’s contrast between decoration and ceramic types… What it does not yet show is that looking at decoration produces more, or different, archaeological interpretation than looking at types or wares alone.”

The reviewer further asked: “Using the same count matrices, show a ware/type-only analysis, a decoration-only analysis, and the joint ware-by-decoration analysis. Then say what conclusion changes when decoration is included.”

Response

I made this the central analytical revision. The earlier and later assemblages are now compared on exactly the same nine-ware × six-technique support at three levels: ware composition (H = 0.430), technique composition (H = 0.588), and joint ware × technique composition (H = 0.734). Because joint H includes both marginal and association differences, I do not treat the difference among these distances as an association effect. A homogeneous-association model therefore tests the relational question directly after aggregate-specific ware and technique margins are fitted. A common ware–technique association is rejected (G² = 118.59; pboot = 0.0001). The added archaeological result is thus not simply that decoration changes, but that decoration is distributed differently among continuing wares after changes in both margins are accounted for.

The manuscript now states explicitly what each analytical level contributes: ware-only analysis shows persistence with shifts in relative ware abundance; technique-only analysis shows the strong movement away from incision/modeling toward stamping/impression; the joint analysis shows that this decorative change is not distributed uniformly across the continuing wares.

Location in revised manuscript: pp. 3–4, lines 42–99; pp. 13–16, lines 301–399; p. 21, lines 488–513; pp. 25–26, lines 592–643.

**Comment 2**

“The paper never defines clearly what it means by a ceramic ‘type’ in relation to a ware and a decoration. This is important because archaeological types may already include decoration, while the ware labels in this paper partly include surface finish.”

Response

The terminology has been rewritten. “Ware” now refers to the recorded macroscopic color-plus-surface-finish categories; “detailed decoration” to the standardized decorative or surface-treatment label assigned to a sherd; “broad technique” to the higher-order analytical grouping; and “type” only to historically published composite constructs such as Burger’s Urabarriu, Chakinani, and Janabarriu categories.

I also explain why a reconstructed Burger-type variable would not provide an independent comparator: those types combine attributes that overlap with the variables analyzed here. Reconstructing the types from the same attributes and then testing them against those attributes would reproduce the circularity raised during review. The recorded ware margin is therefore used as the non-decoration compositional baseline.

Location in revised manuscript: pp. 3–4, lines 42–99; pp. 10–11, lines 224–275.

**Comment 3**

“The Wacheqsa sequence is at once too detailed and not detailed enough. I wanted a clearer high-level orientation before the excavation details.”

Response

I reorganized the opening archaeological section. The Introduction now first locates Wacheqsa relative to the monumental core of Chavín de Huántar, identifies the five stratigraphically differentiated depositional contexts, explains their grouping into earlier and later aggregates, and states immediately that the comparison is neither a five-stage sequence nor a population estimate.

Figure 1 now provides a schematic site and excavation orientation, Figure 2 shows the documented analytical-unit exposures, and Table 1 concentrates the stratigraphic, formation, inventory, and contextual information needed for the comparison.

Location in revised manuscript: pp. 3–9, lines 42–223.

**Comment 4**

“Urabarroid and Janabarroid need to be defined before their first use… A short definition at first use would solve this problem.”

Response

They are now defined in the opening paragraph. Urabarroid denotes Early Platforms + Water Flood; Janabarroid denotes Stone Rooms + Midden + Late Platforms. The -oid suffix indicates ceramic affinity within pre-existing archaeological context sets and does not designate an independently defined phase or ceramic type. The same distinction is repeated in the Methods when the analytical units and aggregate labels are constructed.

Location in revised manuscript: p. 3, lines 42–72; pp. 6–7, lines 130–182.

**Comment 5**

“Much of the excavation volume, artifact-density, and inventory information can move to Table 1 or the supplement. The prose should keep only what is needed for the comparison…”

Response

I condensed the excavation narrative and moved the comparative inventory information into Table 1. The prose now concentrates on stratigraphic position and formation history: the water-laid character of Water Flood, the cumulative-refuse character of Midden, the room-and-alley context of Stone Rooms, and the closure/reorganization character of Late Platforms. The 1945 landslide is mentioned only to explain why interpretation depends on excavated stratigraphy.

Location in revised manuscript: pp. 5–9, lines 100–223; Table 1.

**Comment 6**

“I would also add a compact table in the main text listing the nine shared ware codes and full names, along with the six broad decorative techniques used in the principal comparison.”

Response

Table 2 was added for exactly this purpose. It lists the nine shared ware categories and the six broad techniques used in the principal 9 × 6 common-support analysis, and it states which aggregate-specific categories are excluded from that comparison and why. The complete lexical concordance remains in S1 Table.

Location in revised manuscript: pp. 10–11, lines 224–275; Table 2.

**Comment 7**

“I am less convinced that the evolutionary framework needs so much space… the evolutionary material should be shorter and kept as a statement about questions for future work.”

Response

I shortened and repositioned that discussion. The revised manuscript does not infer learning, drift, selection, conformity, prestige bias, centralized production, or production organization from the observed frequency distributions. Evolutionary concepts are retained only to identify hypotheses that would require independent vessel-level, technological, compositional, spatial, and formation-history evidence.

Location in revised manuscript: pp. 23–25, lines 540–618, especially “What the data do not establish.

**Comment 8**

Reviewer 2 reported that the reproducibility workflow ran successfully and that the validation checks, fixed seeds, session information, reference results, generated figures, and checksum manifest made the numerical analysis easier to assess.

Response

I retained this structure and updated the release to analysis package v12.4 under R 4.5.2. The authoritative workflow now begins from the five analytical-unit matrices, verifies their cellwise aggregation, reproduces the reported analyses and figures, records session information, and runs output validation. From the project root, the complete workflow is executed with:

source("R/00_run_all.R")

Location in revised manuscript: p. 15, lines 350–374; Data Availability, p. 27, lines 644–667.

**Comment 9**

“The OSF project would benefit from a better public front page. It currently has only a one-sentence description, no wiki, no visible license, and no immutable registration… The OSF overview should also identify the manuscript version, explain the difference between the OSF title and the submitted title, map R.zip, the supplementary tables, and the figures to the paper, and give the one-command reproduction route.”

Response

The complete reproducibility package underlying the revised analyses has been permanently archived in the Open Science Framework. The registration contains the authoritative analytical-unit matrices, Supporting Information, generated outputs, and validated R workflow used for the revised manuscript: <https://doi.org/10.17605/OSF.IO/KX9BT>.

**Additional journal requirements**

I have also addressed the technical requirements listed in the decision letter.

**Journal requirement 1 — PLOS ONE style and file naming**

The revised manuscript and accompanying files have been formatted and named in accordance with the PLOS ONE submission requirements. A clean revised manuscript and a version showing tracked changes are included with the resubmission.

**Journal requirement 2 — Ethics statement**

The permits and ethics statement now appears only in the Methods. It states that the Wacheqsa fieldwork was conducted under authorizations issued by the Instituto Nacional de Cultura del Perú in 2003, 2004, and 2005 and that all necessary permits were obtained in compliance with the relevant regulations.

**Journal requirement 3 — Funding Information and Financial Disclosure**

The Funding Information and Financial Disclosure entries have been reconciled in Editorial Manager. The corrected disclosure is: “The author received support for Wacheqsa fieldwork and doctoral research from a Stanford University School of Humanities and Sciences Graduate Research Opportunity Grant, the Stanford Department of Anthropological Sciences, the Stanford Center for Latin American Studies, and the Stanford Archaeology Center. Grant numbers were not assigned. The funders had no role in study design, data analysis, decision to publish, or preparation of the manuscript.

**Journal requirements 4–5 — Figure copyright and permissions**

The copyrighted/proprietary image content identified in the original submission has been removed or replaced. Figure 1 is a newly prepared schematic site map based on project base maps and contains no proprietary map or satellite imagery. Figure 2 derives from the author’s project CAD excavation model. Figure 3 is a newly prepared composite from original Wacheqsa project specimen drawings rather than a reproduction of a previously published third-party figure. The revised Figures 1–3 therefore do not reproduce the proprietary cartographic or third-party published figure content flagged in the original submission.

**Journal requirement 6 — Supporting Information files**

All Supporting Information files cited in the manuscript—S1–S3 Figs and S1–S5 Tables—are included in the Supporting Information package uploaded with the revision.

The review led me to narrow the paper’s claims.

The revised manuscript does not argue that Urabarroid and Janabarroid are two homogeneous ceramic populations, that the Wacheqsa data validate Burger’s site-wide phase sequence, that Chakinani has been disproved, or that a particular social or evolutionary mechanism caused the observed pattern.

The result supported by the present data is more specific. Across the stratigraphically ordered Wacheqsa assemblages, ware composition changes, decorative-technique composition changes, and ware–technique association changes as well, while much of the coded ware repertoire persists. The later contexts also remain internally differentiated.

I believe that this formulation better reflects both the archaeological evidence and the limits of the analytical sample.

Thank you again for the careful reviews and for the opportunity to revise the manuscript.

Christian Mesía-Montenegro
