# Supplementary material for: Changes in ware–decoration associations in ceramic assemblages from Wacheqsa, Chavín de Huántar, Peru
Source: PLoS One. 2026 Oct 29;21(10):e0361889. doi: 10.1371/journal.pone.0361889 (PMC13718927; doi:10.1371/journal.pone.0361889)
Supplement: Supplementary file 3 [file pone.0361889.s005.docx]

Manuscript PONE-D-26-35517R1

Changes in ware–decoration associations in ceramic assemblages from Wacheqsa, Chavín de Huántar, Peru

Dear Dr. Mroczek,

Thank you for the detailed comments. I have clarified the chronological sources, distinguished stratigraphic relationships from contextual interpretation, and expanded the documentation of the analytical samples. Below, I respond to each point and identify the corresponding changes. The numerical results remain those of the validated version 12.4 package.

# 1 Bayesian chronology

**Comment:** The editor requests precise sources for the modeled boundaries, the radiocarbon and contextual evidence underlying the Wacheqsa chronology, and a traceable basis for comparison with architectural reorganization.

Response: I have retained the citation to Mesía-Montenegro and Weber because its published Bayesian model supplies the reported estimates. I now give the exact location: Figure 3, p. 781, and Table 1, p. 782 (revised reference [12]). The End Urabarroid–Start Janabarroid estimate is 878–806 cal BCE and End Janabarroid is 760–581 cal BCE, both at 68.3% probability. Conventional determinations and analytical-unit assignments appear in Mesía-Montenegro (2022), Table 2, p. 887 (revised reference [10]); its Figure 3 shows calibrated determinations, not modeled aggregate boundaries.

Main-text Table 2 lists the ten laboratory determinations, conventional ages and uncertainties, analytical-unit assignments, published modeled intervals, and excavation contexts. The source note identifies the publications and dissertation sections used. I have not recalibrated the dates or fitted a new Bayesian model. The text distinguishes this aggregate chronology from dates of ceramic production or changes in individual decorative practices.

I have retained the architectural comparison as a historical question. Establishing synchronicity or a shared institutional mechanism would require comparison of independently constrained model estimates and their uncertainty across sectors. The ceramic association test does not establish that synchronicity.

Changes: pp. 7–8, lines 156–170, and Table 2; p. 23, lines 540–556, “Stratigraphic order and chronological interpretation.”

# 2 Archaeological contexts and temporal interpretation

**Comment:** The editor asks that direct stratigraphic order be separated from correlations, ceramic affinities, and interpretations of change across deposits with different formation histories.

Response: I have clarified that the five analytical units were constructed before the present frequency analysis using stratigraphic, spatial, architectural, sedimentary, and depositional evidence. Their construction was independent of the standardized frequency matrices, but was not blind to all archaeological material.

I now distinguish local superposition from cross-exposure correlation. Stone Rooms overlies Early Platforms in several exposures; the relationship between WQ-4 layer 6 and the Stone Rooms network represented by WQ-8 layer 3 involves correlation between exposures. Midden overlies or cuts Water Flood in southern WQ-7. The Early Platforms–Late Platforms comparison is qualified because the deeper Late Platforms determination AA75388 was already recognized as potentially overlapping the earlier occupation. Table 7 now acknowledges this qualification.

The Discussion treats the units as distinct activity and depositional settings. It retains an archaeological interpretation of differing decorative repertoires while recognizing that elapsed time cannot be separated from activity composition, use, disposal, and reworking. A continuous or equally spaced five-stage sequence is not inferred.

Changes: pp. 6–7, lines 121–153; Table 7; pp. 22–23, lines 503–556.

# 3 Unequal samples and direct context comparisons

**Comment:** The editor requests greater interpretive use of individual context comparisons and continued caution about the small Water Flood sample.

**Response:** The numerical imbalance remains explicit: Early Platforms contributes 330 of 352 earlier observations (93.8%), and Midden contributes 691 of 956 later observations (72.3%). These are shares of the analytical sample, not estimates of archaeological prevalence.

Tables 6 and 7 support direct comparisons of Early Platforms with Stone Rooms, Midden, and Late Platforms. Stamping/impression comprises 6/330 observations in Early Platforms (1.8%), 117/215 in Stone Rooms (54.4%), 456/691 in Midden (66.0%), and 34/50 in Late Platforms (68.0%). Its prominence is therefore not confined to the largest refuse sample. I also discuss differences among the later joint profiles rather than treating the pooled Janabarroid aggregate as homogeneous.

The leave-Midden-out comparison checks the influence of the largest later sample; it does not make the remaining deposits functionally equivalent. Water Flood’s 27.3% stamping/impression represents six observations out of 22. This small, water-laid sample cannot establish a transitional stage. I also qualify the interpretation of the small Late Platforms sample.

Changes: p. 11, lines 267–270; Tables 6 and 7; pp. 21–22, lines 490–513.

# 4 Reduction to common support

**Comment:** The editor requests explicit accounting for the differing exclusion proportions and discussion of the shared-support and union-support distances.

**Response:** Table 4 now gives mutually exclusive exclusion categories. In the earlier aggregate, two observations are excluded for ware alone, fifteen for technique alone, and one for both: eighteen of 352 (5.1%), leaving 334. In the later aggregate, the corresponding counts are 67, 66, and one: 134 of 956 (14.0%), leaving 822. The text also explains the sequential filter, which removes three and 68 observations for aggregate-specific wares before excluding fifteen and 66 for technique among the retained wares.

The joint Hellinger distance is H = 0.734 on shared support, H = 0.752 with all nine broad classes retained within the shared wares, and H = 0.763 on full union support. Retaining the excluded categories modestly increases the descriptive contrast. Because the supports and renormalized profiles differ, this increase cannot be assigned as an additive contribution of particular excluded categories.

Changes: p. 11, Table 4 and lines 261–265; pp. 18–19, lines 425–450; p. 24, lines 564–567; S4 Table.

# 5 Original coding procedure

**Comment:** The editor asks what is known about the original single-label procedure, what cannot be recovered, and how those gaps could affect the association analysis.

Response: I have specified what survives: one decoration or surface-treatment label per sherd, with matrices and a concordance documenting how those labels were grouped into broad classes. Co-occurring treatments and the original rule for choosing among them are not preserved. Procedures for eroded or ambiguous specimens and observer-agreement checks are undocumented.

A treatment-priority rule could affect recorded ware–technique frequencies, even if applied consistently, when treatment co-occurrence differed among wares or contexts. Without the additional treatments or original selection rule, this effect cannot be quantified. The analysis therefore concerns recorded labels rather than the complete distribution of treatments on individual sherds. I have not retrospectively reclassified them.

Changes: p. 10, lines 234–248; p. 24, lines 558–563; S1 Table.

# 6 Meaning of the homogeneous association test

**Comment:** The editor requests explicit limits on temporal attribution and vessel independence, and greater emphasis on actual ware–technique combinations.

Response: I have clarified that model rejection does not identify an archaeological mechanism. The homogeneous-association test evaluates a common association after fitting aggregate-specific margins. Its bootstrap calibration applies to the fitted sherd-count model; it does not account for unknown clustering of fragments within vessels or reconstruct the original physical sampling design.

I now emphasize the actual combinations. Within retained Gray Polished observations, incision changes from 89.4% to 5.3%, while stamping/impression changes from 0% to 86.0%. Incision changes from 93.3% to 12.4% in Black Polished and from 80.0% to 11.2% in Brown Polished; stamping/impression changes from 4.4% to 81.1% and from 8.0% to 84.1%, respectively. These are within-ware percentages, not overall ware abundances.

The interpretation relates these combinations to documented activity settings, collective consumption, and possible movement into refuse and construction fills. A role for decoration in serving or presentation remains a contextual hypothesis. The matrices alone cannot establish that role or demonstrate rank, affiliation, ritual authority, or institutional transformation.

Changes: pp. 13–14, lines 312–336; pp. 15–19, lines 365–450; pp. 20–22, lines 468–539; p. 24, lines 558–573; Fig 5 and S5 Table.

# 7 Illustrated specimen identification

**Comment:** The editor requests specimen identifiers and links between each Figure 3 drawing, excavation context, and curated collection, or a precise explanation of the surviving documentation.

Response: Figure 3 has been updated to show the recorded excavation provenience for each of its 19 illustrated sherds, including excavation unit, level and, where applicable, sector and subunit. Panel A derives from Early Platforms; in panel B, a–c, e–g and j derive from Midden, while d, h and i derive from Stone Rooms. These locators substantially improve the traceability of the illustrations but are not unique specimen or museum inventory numbers. The archaeological collection is curated at the Museo Nacional de Chavín, Ancash, Peru. A unique drawing-to-object concordance is not preserved in the analytical archive; identifying an individual object would require checking the original drawing sheets against the collection records.

Changes: p. 10, Fig 3 and caption, lines 215–232.

# 8 Fieldwork authorizations

**Comment:** The editor requests reference numbers for the 2003–2005 authorizations.

**Response:** The Permits and Ethics section now gives the Instituto Nacional de Cultura del Perú authorizations: Resolución Directoral Nacional Nº 824/INC, 4 August 2003; Nº 704/INC, 23 August 2004; and Nº 902/INC, 4 July 2005. The statement also distinguishes the original fieldwork from the present analysis of previously classified material, which involved no new excavation, destructive sampling, or export.

Changes: p. 15, lines 353–362, “Permits and ethics.”

# 9 References

**Comment:** The editor requests sequential numbering, removal or appropriate citation of unused references, correction of the 2014 title, and clarification of the Tello archival source.

Response: I have renumbered the references in order of first citation. All 24 entries are cited in the text; previously unused entries have been cited where relevant or removed. The Wacheqsa Bayesian estimates are attributed to the published model that produced them.

The 2014 title now includes “Período Formativo Tardío.” I have removed the Tello archival citation. Sources for the sector’s archaeological and chronological background are cited where used.

Changes: citations throughout the manuscript; references on pp. 26–27, including revised reference [22].

# Data availability and reproducibility

**Comment:** The editor requests confirmation that the final deposit is the package used for the revised outputs and that the documented command reproduces the analysis.

**Response:** The final analysis package, version 12.4, is deposited at DOI 10.17605/OSF.IO/KX9BT. I successfully executed source("R/00_run_all.R") from the package root and verified that the generated outputs agree with the numerical results reported in the revised tables and figures. The deposit contains the five unit-level matrices, classification concordance, scripts, generated outputs, bootstrap distributions, and execution/validation records.

Supporting Tables S1–S5 document the classification, aggregate and unit matrices, correspondence analysis, Hellinger comparisons, and homogeneous-association diagnostics. Chronological source information is in main-text Table 2, and specimen documentation is addressed in the Figure 3 caption.

Changes: p. 15, lines 346–351, “Reproducibility”; p. 25, lines 588–599, “Data availability”; supporting-information captions.

# Funding disclosure

**Comment:** The editor asks whether the National Geographic Society and the Global Heritage Fund supported the reported work and requests consistency across the manuscript, cover letter, and Financial Disclosure field.

**Response:** The revised declaration identifies Stanford University, the National Geographic Society, and the Global Heritage Fund as supporters of the Wacheqsa fieldwork and doctoral research. It specifies Stanford’s contributing units and the Graduate Research Opportunity Grant, as well as the Global Heritage Fund’s provision of Autodesk Land and Autodesk Map software licenses.

The declaration states that no grant numbers were assigned and that the funders had no role in study design, data analysis, the decision to publish, or manuscript preparation. The same declaration appears in the revised manuscript and cover letter. The Financial Disclosure field will be checked for consistency during resubmission. If correction of the existing entry requires assistance from the journal office, I request that it be updated accordingly.

Changes: p. 25, lines 604–609, “Funding.”

# Title

**Comment:** The editor suggests a shorter title describing assemblage comparisons without implying a continuous sequence.

Response: I have adopted the suggested title: “Changes in ware–decoration associations in ceramic assemblages from Wacheqsa, Chavín de Huántar, Peru.”

Change: title page.

# Reviewer 2

**Comment:** Reviewer 2 considers the previous concerns addressed and recommends acceptance.

Response: I thank Reviewer 2 for the assessment and recommendation. This revision addresses the Academic Editor’s additional requests while retaining the numerical results and strengthening their archaeological interpretation.

Sincerely,

Christian Mesía-Montenegro
