# Supplementary material for: Changes in ware–decoration associations in ceramic assemblages from Wacheqsa, Chavín de Huántar, Peru
Source: PLoS One. 2026 Oct 29;21(10):e0361889. doi: 10.1371/journal.pone.0361889 (PMC13718927; doi:10.1371/journal.pone.0361889)
Supplement: Peer review history

# Peer review: Decision Letter 0

**DOI:** 10.1371/journal.pone.0361889.r001

**Authors:** Przemysław Mroczek

13 Sep 2026

PLOS One

Dear Dr. Mesia-Montenegro,

Thank you for submitting your manuscript to PLOS One. After careful consideration, we feel that it has merit but does not fully meet PLOS One’s publication criteria as it currently stands. Therefore, we invite you to submit a revised version of the manuscript that addresses the points raised during the review process.

=============================

The reviewers differ in their recommendations, but they raise several of the same underlying concerns. I am prepared to allow a major revision, in line with Reviewer 2, but the revised manuscript must address Reviewer 1’s concerns about circularity, archaeological framing and the meaning of “continuity with redistribution”; it should also clarify the relationship among type, ware, decoration, Urabarroid and Janabarroid, and show what the joint ware–decoration analysis contributes beyond separate analyses of ware and decoration. The interpretation should remain within the limits of the uneven analytical units and the confounding of chronology, activity and formation processes; I also recommend a clearer presentation of the Wacheqsa context and a shorter evolutionary discussion unless it can be linked directly to predictions tested by the data.

plosone@plos.org. When you're ready to submit your revision, log on to https://www.editorialmanager.com/pone/ and select the 'Submissions Needing Revision' folder to locate your manuscript file.

- A letter that responds to each point raised by the academic editor and reviewer(s). You should upload this letter as a separate file labeled 'Response to Reviewers'.
- A marked-up copy of your manuscript that highlights changes made to the original version. You should upload this as a separate file labeled 'Revised Manuscript with Track Changes'.
- An unmarked version of your revised paper without tracked changes. You should upload this as a separate file labeled 'Manuscript'.

If you would like to make changes to your financial disclosure, please include your updated statement in your cover letter. Guidelines for resubmitting your figure files are available below the reviewer comments at the end of this letter.

If applicable, we recommend that you deposit your laboratory protocols in protocols.io to enhance the reproducibility of your results. Protocols.io assigns your protocol its own identifier (DOI) so that it can be cited independently in the future. For instructions see: https://journals.plos.org/plosone/s/submission-guidelines#loc-laboratory-protocols. Additionally, PLOS One offers an option for publishing peer-reviewed Lab Protocol articles, which describe protocols hosted on protocols.io. Read more information on sharing protocols at https://plos.org/protocols?utm_medium=editorial-email&utm_source=authorletters&utm_campaign=protocols.

As the corresponding author, your ORCID iD is verified in the submission system and will appear in the published article. PLOS supports the use of ORCID, and we encourage all coauthors to register for an ORCID iD and use it as well. Please encourage your coauthors to verify their ORCID iD within the submission system before final acceptance, as unverified ORCID iDs will not appear in the published article. *Only*  the individual author can complete the verification step; PLOS staff *cannot*  verify ORCID iDs on behalf of authors.

We look forward to receiving your revised manuscript.

Kind regards,

Przemysław Mroczek, Dr. hab.

Academic Editor

PLOS One

Journal Requirements:

When submitting your revision, we need you to address these additional requirements.

1. Please ensure that your manuscript meets PLOS ONE's style requirements, including those for file naming. The PLOS ONE style templates can be found at

https://journals.plos.org/plosone/s/file?id=wjVg/PLOSOne_formatting_sample_main_body.pdf and https://journals.plos.org/plosone/s/file?id=ba62/PLOSOne_formatting_sample_title_authors_affiliations.pdf

2. Your ethics statement should only appear in the Methods section of your manuscript. If your ethics statement is written in any section besides the Methods, please delete it from any other section.

3. We note that the grant information you provided in the ‘Funding Information’ and ‘Financial Disclosure’ sections do not match.

When you resubmit, please ensure that you provide the correct grant numbers for the awards you received for your study in the ‘Funding Information’ section.

4. We note that Figure 1 in your submission contain [map/satellite] images which may be copyrighted. All PLOS content is published under the Creative Commons Attribution License (CC BY 4.0), which means that the manuscript, images, and Supporting Information files will be freely available online, and any third party is permitted to access, download, copy, distribute, and use these materials in any way, even commercially, with proper attribution. For these reasons, we cannot publish previously copyrighted maps or satellite images created using proprietary data, such as Google software (Google Maps, Street View, and Earth). For more information, see our copyright guidelines: http://journals.plos.org/plosone/s/licenses-and-copyright.

We require you to either (1) present written permission from the copyright holder to publish these figures specifically under the CC BY 4.0 license, or (2) remove the figures from your submission:

a. You may seek permission from the original copyright holder of Figure 1 to publish the content specifically under the CC BY 4.0 license.

We recommend that you contact the original copyright holder with the Content Permission Form (http://journals.plos.org/plosone/s/file?id=7c09/content-permission-form.pdf) and the following text:

“I request permission for the open-access journal PLOS ONE to publish XXX under the Creative Commons Attribution License (CCAL) CC BY 4.0 (http://creativecommons.org/licenses/by/4.0/). Please be aware that this license allows unrestricted use and distribution, even commercially, by third parties. Please reply and provide explicit written permission to publish XXX under a CC BY license and complete the attached form.”

Please upload the completed Content Permission Form or other proof of granted permissions as an "Other" file with your submission.

In the figure caption of the copyrighted figure, please include the following text: “Reprinted from [ref] under a CC BY license, with permission from [name of publisher], original copyright [original copyright year].”

b. If you are unable to obtain permission from the original copyright holder to publish these figures under the CC BY 4.0 license or if the copyright holder’s requirements are incompatible with the CC BY 4.0 license, please either i) remove the figure or ii) supply a replacement figure that complies with the CC BY 4.0 license. Please check copyright information on all replacement figures and update the figure caption with source information. If applicable, please specify in the figure caption text when a figure is similar but not identical to the original image and is therefore for illustrative purposes only.

The following resources for replacing copyrighted map figures may be helpful:

USGS National Map Viewer (public domain): http://viewer.nationalmap.gov/viewer/

The Gateway to Astronaut Photography of Earth (public domain): http://eol.jsc.nasa.gov/sseop/clickmap/

Maps at the CIA (public domain): https://www.cia.gov/library/publications/the-world-factbook/index.html and https://www.cia.gov/library/publications/cia-maps-publications/index.html

NASA Earth Observatory (public domain): http://earthobservatory.nasa.gov/

Landsat: http://landsat.visibleearth.nasa.gov/

USGS EROS (Earth Resources Observatory and Science (EROS) Center) (public domain): http://eros.usgs.gov/#

Natural Earth (public domain): http://www.naturalearthdata.com/

5. We note that Figure(s) 1, 2, 3 in your submission contain copyrighted images. All PLOS content is published under the Creative Commons Attribution License (CC BY 4.0), which means that the manuscript, images, and Supporting Information files will be freely available online, and any third party is permitted to access, download, copy, distribute, and use these materials in any way, even commercially, with proper attribution. For more information, see our copyright guidelines: http://journals.plos.org/plosone/s/licenses-and-copyright.

We require you to either (1) present written permission from the copyright holder to publish these figures specifically under the CC BY 4.0 license, or (2) remove the figures from your submission:

a. You may seek permission from the original copyright holder of Figure(s) 1, 2, 3 to publish the content specifically under the CC BY 4.0 license.

We recommend that you contact the original copyright holder with the Content Permission Form (http://journals.plos.org/plosone/s/file?id=7c09/content-permission-form.pdf) and the following text:

“I request permission for the open-access journal PLOS ONE to publish XXX under the Creative Commons Attribution License (CCAL) CC BY 4.0 (http://creativecommons.org/licenses/by/4.0/). Please be aware that this license allows unrestricted use and distribution, even commercially, by third parties. Please reply and provide explicit written permission to publish XXX under a CC BY license and complete the attached form.”

Please upload the completed Content Permission Form or other proof of granted permissions as an "Other" file with your submission.

In the figure caption of the copyrighted figure, please include the following text: “Reprinted from [ref] under a CC BY license, with permission from [name of publisher], original copyright [original copyright year].”

b. If you are unable to obtain permission from the original copyright holder to publish these figures under the CC BY 4.0 license or if the copyright holder’s requirements are incompatible with the CC BY 4.0 license, please either i) remove the figure or ii) supply a replacement figure that complies with the CC BY 4.0 license. Please check copyright information on all replacement figures and update the figure caption with source information. If applicable, please specify in the figure caption text when a figure is similar but not identical to the original image and is therefore for illustrative purposes only.

6. Please upload a copy of Supporting Information Figure/Table. S1 Fig, S2 Fig, S3 Fig, S1 Table, S2 Table, S3 Table, S4 Table, S5 Table, which you refer to in your text on page 39.

7. If the reviewer comments include a recommendation to cite specific previously published works, please review and evaluate these publications to determine whether they are relevant and should be cited. There is no requirement to cite these works unless the editor has indicated otherwise.

Additional Editor Comments :

Reviewer 1 recommends rejection, mainly because the paper does not yet make a convincing archaeological case for the analysis. The reviewer questions the purpose of the study, the meaning of “continuity with redistribution”, and the risk of circularity when historically defined ceramic groupings are compared using ceramic attributes. Reviewer 2 recommends major revision and is more positive about the statistical analysis and reproducibility, but raises a related problem: the manuscript does not yet show what the joint ware–decoration analysis adds beyond analysing ware and decoration separately. Both reviewers also point to unclear terminology and problems with the presentation of the archaeological context.

I have read the manuscript alongside both reports. The present version is not ready for publication, but I think the main problems can be addressed without new fieldwork or new archaeological material. I am therefore inviting a major revision.

The main issue is the contribution of the joint ware-decoration analysis. The revised manuscript should compare, using the existing dataset, ware-only composition, decoration-only composition and joint ware × decoration structure. The author should then state clearly what is learned from the joint analysis that is not already apparent from the two marginal patterns. If the joint analysis does not change the archaeological interpretation, the claims should be reduced accordingly.

The terminology also needs revision. “Type”, “ware”, “decoration”, “technique”, Urabarroid and Janabarroid should be defined clearly and used consistently. The manuscript should distinguish the stratigraphic and contextual basis of the five analytical units from the ceramic affinities expressed by the Urabarroid and Janabarroid labels. This distinction is necessary to address the concern about circular reasoning and to avoid giving the impression that the analysis independently validates the traditional Urabarriu–Chakinani–Janabarriu sequence.

The limits of the dataset also need to remain visible in the interpretation. The earlier aggregate is dominated by Early Platforms and the later aggregate by Midden. The leave-Midden-out test shows that the main numerical pattern is not caused by that sample alone, but it does not separate chronology from activity, depositional context or formation processes. Statements about changes through time should therefore be made cautiously and should refer to the analysed sherd assemblages rather than to vessel populations or ceramic production more generally.

The archaeological context should be presented more directly. Wacheqsa, the five analytical units and their grouping into earlier and later assemblages should be explained before the detailed analytical discussion. Urabarroid and Janabarroid should be defined at first use. The evolutionary discussion can also be shortened unless the author can link it to predictions that are actually tested by the present data.

I am giving the author an opportunity to revise the manuscript rather than rejecting it at this stage. This is not an indication of likely acceptance. The revised version will need to address the concerns raised by both reviewers, particularly the purpose of the joint ware-decoration analysis, the possible circularity of the classification framework, and the limits placed on interpretation by the structure of the archaeological sample. A point-by-point response should accompany the revision.

[Note: HTML markup is below. Please do not edit.]

Reviewers' comments:

Reviewer's Responses to Questions

**Comments to the Author**

1. Is the manuscript technically sound, and do the data support the conclusions?

Reviewer #1: Partly

Reviewer #2: Yes

**********

2. Has the statistical analysis been performed appropriately and rigorously?

Reviewer #1: I Don't Know

Reviewer #2: Yes

**********

3. Have the authors made all data underlying the findings in their manuscript fully available?

The PLOS Data policy

Reviewer #1: Yes

Reviewer #2: Yes

**********

4. Is the manuscript presented in an intelligible fashion and written in standard English?

Reviewer #1: No

Reviewer #2: No

**********

Reviewer #1: This paper is an attempt at analyzing the changes in different attributes (both technological and decorative) in ceramic types at Wacheqsa, a sector of the famous site of Chavin de Huantar (1100-400 BCE) in the highlands of Peru. The ceramic data (i.e., sherds) analyzed come from archaeological excavations carried out between 2003-2005 that sampled 132 sq m of the Wacheqsa sector. Based on architectural and stratigraphic evidence, the author identifies five occupation phases at Wacheqsa (i.e., the "five analytical units").

The analysis presented in this MS focuses on contrasting what archaeologists have called "types" (aka "type varieties"): Urabarriu and Janabarriu, originally defined by Richard Burger in the late 1970s and 1980s with the objective of chronologically ordering the occupation sequence at Chavin de Huantar. These were originally part of a tripartite typology that also included a Chakinani type thought to be chronologically squeezed between Urabarriu and Janabarriu but which archaeologists seem to be questioning. In the current MS, the author considers “Urabarriu" is associated with an earlier occupation (dated in this MS to 878-806 cal BCE) and Janabarriu with a later occupation (dated in this MS to 760-581 cal BCE).

The gist of my commentary goes as follow: the ceramics and archaeological data more generally are welcome, but it is not clear to me what the goal of the MS is. Is it a methodological contribution? Or one that hopes to contribute to Chavin studies and the anthropological meaning of changes in pottery making practices during the first millennium BCE at Chavin de Huantar? Where are the humans (i.e., the anthropology)?

The remainder of my review will try to unpack how I think so and that I think can be done to improve the MS. I hope that my narrative will not be too repetitive, but I do circle back often to some key points. I am trying to be as constructive and generous as I can.

The writing needs significant reworking for clarity, but I was able to surmise that the author is comparing sherds from the two types (n=352 Urabarriu sherds + n=956 Janabarriu sherds for a total of n=1308 sherds) so as to identify and understand potential changes in pottery making practices including technological and decorative techniques. Through various statistical tools (e.g., Pearson's chi-square, Hellinger distance), the author suggests that both "types" share attributes, which the author interprets as meaning that Janabarriu did not replace "wholesale" Urabarriu. Considering that types are analytical tools created and used by cultural historians to control time and name “archaeological phases or cultures” (not people!), this observation has little value (and could even be perceived as “strawman” or “red herring” to some extent). Also, to me the statistical tools appear overkill but I am not a statistician and for reasons that should become clear below, think that the author is simply asking the wrong questions (or questions to which we seem to already have the answers to [i.e., that Urabarriu and Janabarriu are different, but not completely]).

I gather that the author's "decorative categories" refer to what archaeologists call "decorative style" (e.g., surface treatment, motifs), while "wares" refer to what archaeologists typically refer to as "technological style" (e.g., manufacturing techniques, materials). The author concludes that the "overlap" in attributes between Urabarriu and Janabarriu ceramic samples represents a pattern of "continuity with redistribution," although it is unclear to me what that means in anthropological and archaeological terms. Does it mean that attributes show continuity across time as seen in the two types, but that they are redistributed within the types? Or rather that each type or attributes are spatially (re?)distributed through time? What does “redistribution” even mean? What is the different between “distribution” and “redistribution”? It seems to me that the distribution of certain types of archaeological materials at a given site might be impacted by several factors, including taphonomical (i.e., discard, preservation, N-transforms, C-transforms, your basic Schifferian view) and archaeological (i.e., recovery, sampling, screening, looking for “diagnostics”) ones. Is the analysis an attempt at mapping and understanding these factors? Or rather, is the author talking about the distribution of traits/features/attributes within each type (which would inform on the practice of pottery making itself)?

In any case, the author concludes that Urabarriu and Janabarriu are best treated as "local families of stratified assemblages rather than internally uniform, site-wide ceramic types." I suppose that this means something to statisticians, but what does it means in anthropological and/or archaeological terms? Aren't archaeologists studying the people who made the pots?

While I commend the author for trying to apply statistical tools to analyze the dataset, I do not see how the analysis brings heuristic value to our understanding of Chavin pottery making. Archaeologists study and analyze material remains to understand humans, their actions, societies, relations, etc. Analyzing the structure of archaeological samples/assemblages in an anthropological or historical vacuum, just for the sake of it, appears of limited value -- except if the goal of the paper is explicitly to make a methodological contribution (which perhaps it is!).

Perhaps that with a more solid contextualization of pottery making and Chavin studies, as well as the ancient occupation of Chavin de Huantar, the paper would make more sense (although the author makes it clear that the goal of the MS is not to extrapolate to the site of Chavin de Huantar [lines 198-200] so one wonders then what the goal of the MS is: is it purely a methodological contribution? In other words, testing the analytical value of certain statistical tools? If not, and if the paper is trying to make a contribution to Chavin studies, the main problem seems to be the lack of anthropological and archaeological contextualization of the study beyond the very restricted scope of the ceramic assemblage at hand.)

To me, the analysis presented amounts to an exercise in circular reasoning: Urabarriu and Janabarriu were defined within a cultural historical framework and the explicit goal of ordering time and sequencing the ancient occupation at Chavin de Huantar. The archaeologist who suggested this seriation focused on key differences (i.e., attributes that varied) to distinguish the two types and relied on stratigraphic data to order the seriation (i.e., Urabarriu is earlier than Janabarriu). We already know this and we also know that both types share many attributes. The goal of culture history and seriation is to emphasize differences so as to effectively rely on so-called "diagnostic" traits to classify materials, ideally in phases, periods, etc. Again, it is unclear to me what the proposed analysis brings to that debate.

To rephrase, archaeologists are well aware that type seriations are ultimately analytical exercises that will always have some levels of arbitrariness. The types themselves do not exist "out there" and they would not have meant anything to the potters themselves some 3000 years ago. The question then becomes, what is the goal of the author here? Demonstrate that Urabarriu and Janabarriu share some but not all technological and decorative "attributes"? We already know this! That sherds categorized in each type found in different proportions/frequencies in different contexts (stratigraphically or spatially within a given occupation phase)? If so, why not present distribution maps, stratigraphic sections and more data on the archaeological contexts themselves?

Considering that the author seems to have access to very rich contextual data from the excavations, I fail to see how innovative or original the statistical treatment is to "characterize" and "order" Urabarriu vs. Janabarriu pottery types. On the other hand, what seems of value might be to try to understand how and why ceramic technologies and decorative tastes change over time, something that is not really addressed in this MS (except for a few lines in the conclusions, but even then the "evolutionary hypotheses" raised by the author which I do not see as anthropologically informed). I realize that this is not what the author has in mind, as indicated in the last sentence of the Conclusions (lines 697-699): "The present contribution is the reproducible quantitative baseline..."

My recommendation would be for the author to either (1) present contextual and ceramic data to contribute to Chavin studies and our understanding of pottery making or (2) explicitly frame the MS in methodological terms, with a proper review of how the analysis contributes to ceramic analytical methods (readers will need to see how the proposed methods add to traditional type seriation, attribute analysis, decorative stylistic, and technological stylistic approach).

Reviewer #2: The manuscript is generally written in clear, standard English. I did not identify pervasive grammatical or typographical problems requiring copyediting. However, readability would improve substantially with revision of the organization and terminology. In particular, the opening Wacheqsa context needs to orient readers before presenting excavation detail; “Urabarroid” and “Janabarroid” should be defined before first use; and a compact table of the shared wares and broad decorative techniques would make the analytical sections easier to follow. At lines 66 and 86, “the monumental core” should be specified as “the monumental core of Chavin de Huantar.”

**********

what does this mean?). If published, this will include your full peer review and any attached files.

If you choose “no”, your identity will remain anonymous but your review may still be made public.

**Do you want your identity to be public for this peer review?**  For information about this choice, including consent withdrawal, please see our Privacy Policy

Reviewer #1: No

Reviewer #2: No

**********

[NOTE: If reviewer comments were submitted as an attachment file, they will be attached to this email and accessible via the submission site. Please log into your account, locate the manuscript record, and check for the action link "View Attachments". If this link does not appear, there are no attachment files.]

To ensure your figures meet our technical requirements, please review our figure guidelines: https://journals.plos.org/plosone/s/figures

You may also use PLOS’s free figure tool, NAAS, to help you prepare publication quality figures: https://journals.plos.org/plosone/s/figures#loc-tools-for-figure-preparation.

NAAS will assess whether your figures meet our technical requirements by comparing each figure against our figure specifications.

Attachment Submitted filename: PONE-D-26-35517_review.pdf

# Author comment: Author response to Decision Letter 1

**DOI:** 10.1371/journal.pone.0361889.r002

22 Sep 2026

Response to the Academic Editor and Reviewers

Manuscript PONE-D-26-35517R1

Ceramic change at Wacheqsa, Chavín de Huántar: Changing associations between ware and decorative technique in an Andean Formative assemblage

Dear Dr. Mroczek and Reviewers,

Thank you for the time invested in evaluating the manuscript and for giving me the opportunity to revise it. I took the concerns raised in the decision letter and the two reports as requiring changes to the argument, not simply additions to the text.

The most substantial revision concerns the purpose of the joint ware–decoration analysis. The paper now compares ware composition, technique composition, and joint ware × technique composition on exactly the same analytical support, and then tests separately whether ware–technique association changes after differences in the two margins have been fitted. I have also rewritten the terminology and the treatment of the inherited ceramic sequence, expanded the archaeological presentation of Wacheqsa and the five analytical units, made the sample structure and inferential limits explicit throughout, shortened the evolutionary discussion, and rebuilt the Supporting Information and reproducibility package around the final unit-derived analysis.

The revised manuscript is cited below by page and numbered manuscript lines.

Academic Editor — Dr. Przemysław Mroczek

Comment 1

“The main issue is the contribution of the joint ware-decoration analysis. The revised manuscript should compare, using the existing dataset, ware-only composition, decoration-only composition and joint ware × decoration structure. The author should then state clearly what is learned from the joint analysis that is not already apparent from the two marginal patterns.”

Response

I agree. This became the central analytical revision.

The earlier and later assemblages are now compared on identical support comprising nine wares and six broad techniques. Three Hellinger comparisons are reported separately:

ware composition, H = 0.430 (95% fixed-count interval 0.392–0.481); technique composition, H = 0.588 (0.551–0.634); and joint ware × technique composition, H = 0.734 (0.714–0.793).

I also make explicit that these three distances are not additive. In particular, the larger joint H is not interpreted as an association effect. Joint H can incorporate both marginal change and association change.

For that reason, I added a boundary-aware homogeneous-association log-linear model. The model allows the two aggregates to have different ware margins and different technique margins while constraining their ware–technique association to be common. That model is rejected (G² = 118.59; effective residual dimension = 28; pboot = 0.0001). All 9,999 parametric-bootstrap refits converged, with no simulated deviance reaching the observed value.

The added information from the joint analysis is therefore now stated precisely: the later assemblage differs not only in the abundance of wares and techniques, but also in the way decorative techniques are distributed among the continuing ware categories.

This distinction now structures the Introduction, Methods, Results, Discussion, and Conclusions.

Location in revised manuscript: pp. 3–4, lines 42–99; pp. 13–14, lines 301–349; pp. 15–16, lines 350–399; p. 21, lines 488–513; pp. 25–26, lines 592–643. The final Methods explicitly distinguishes the six Hellinger estimands and states why association heterogeneity is tested separately. The principal Results report the three marginal/joint distances and the association-specific model.

Comment 2

“The terminology also needs revision. ‘Type’, ‘ware’, ‘decoration’, ‘technique’, Urabarroid and Janabarroid should be defined clearly and used consistently.”

Response

I revised the terminology throughout the paper.

In the revised manuscript, ware denotes the macroscopic color-plus-surface-finish categories recorded in the surviving matrices. Detailed decoration denotes the standardized decorative or surface-treatment label assigned to a sherd. Broad technique is the higher-order analytical grouping used in the comparative analysis. Type is reserved for historically published composite typological constructs such as Urabarriu, Chakinani, and Janabarriu.

Urabarroid and Janabarroid are defined at first use as local Wacheqsa aggregate labels. Urabarroid denotes Early Platforms + Water Flood; Janabarroid denotes Stone Rooms + Midden + Late Platforms. The -oid suffix indicates ceramic affinity and does not designate an independently defined phase or ceramic type.

I also clarify that ‘ware’ in this dataset is an operational macroscopic color-plus-surface-finish category. I do not use it as a synonym for technological style or as a direct measure of manufacturing technology, paste recipe, or production organization

The same terminology is used consistently in the Methods, Results, tables, figures, and Discussion.

Location in revised manuscript: p. 3, lines 42–72; pp. 6–7, lines 130–182; pp. 10–11, lines 224–275. The operational definitions are stated explicitly in the Methods.

Comment 3

“The manuscript should distinguish the stratigraphic and contextual basis of the five analytical units from the ceramic affinities expressed by the Urabarroid and Janabarroid labels. This distinction is necessary to address the concern about circular reasoning and to avoid giving the impression that the analysis independently validates the traditional Urabarriu–Chakinani–Janabarriu sequence.”

Response

I agree that the earlier manuscript did not make this distinction clearly enough.

The revised paper now separates the archaeological context sets from the ceramic-affinity labels attached to them.

The five analytical units were constructed during the original post-excavation analysis from stratigraphic position, spatial continuity, sediment matrix, deposit geometry, architectural association, and formation history. Their principal stratigraphic relationships therefore predate the standardized ware–decoration matrices analyzed in this paper.

Ceramic affinity subsequently motivated the descriptive labels Urabarroid and Janabarroid, but those labels did not create the five context sets or their principal stratigraphic ordering.

I also explain why Burger’s types were not reconstructed as another analytical variable. Those historical types combine attributes that include finish, form, and decoration. Reconstructing a Burger-type assignment from the same attributes subsequently entered into the analysis would not provide an independent comparator. The revised paper therefore uses the recorded ware margin as the non-decoration compositional baseline.

The manuscript no longer presents its results as an independent validation of Burger’s phase sequence.

Location in revised manuscript: pp. 3–4, lines 42–99; pp. 5–8, lines 100–206; pp. 24–25, lines 566–618. The construction of the units and their relationship to the -oid labels are now described directly.

Comment 4

“The limits of the dataset also need to remain visible in the interpretation. The earlier aggregate is dominated by Early Platforms and the later aggregate by Midden. The leave-Midden-out test shows that the main numerical pattern is not caused by that sample alone, but it does not separate chronology from activity, depositional context or formation processes.”

Response

This limitation is now stated throughout the manuscript rather than only in the Discussion.

Early Platforms contributes 330 of 352 observations (93.8%) to the earlier analytical sample. Midden contributes 691 of 956 observations (72.3%) to the later sample. I therefore do not treat the aggregate contrast as balanced replication of two chronological populations.

The leave-Midden-out comparison is now reported alongside the principal aggregate result. After Midden is removed, ware H decreases from 0.430 to 0.352, technique H from 0.588 to 0.560, while joint H remains 0.733. The homogeneous-association model continues to reject a common association (G² = 90.16; effective residual dimension = 24; pboot = 0.0001).

The interpretation has been deliberately restricted. The sensitivity analysis shows that the principal association result is not produced solely by Midden. It does not remove confounding among chronology, activity composition, formation process, fragmentation, preservation, or recovery. Water Flood is also too small to provide a genuinely independent earlier replicate.

Location in revised manuscript: pp. 9–12, lines 207–300; pp. 15–16, lines 350–399; pp. 21 and 25–26, lines 488–513 and 592–643. The scope-of-inference section now states these restrictions before the inferential analyses are presented. The Results retain the same qualification after the leave-Midden-out test.

Comment 5

“Statements about changes through time should therefore be made cautiously and should refer to the analysed sherd assemblages rather than to vessel populations or ceramic production more generally.”

Response

I revised the language accordingly.

The observational unit is now stated explicitly as one classified sherd. The manuscript notes that systematic vessel refitting was not undertaken or is not retained in the analytical record, so the observations cannot be treated as independent vessel counts.

Fixed-count resampling and Dirichlet sensitivity ranges are therefore described as uncertainty or sensitivity of the analyzed sherd profiles, not of vessel populations. The conclusions are likewise restricted to stratigraphically ordered Wacheqsa sherd assemblages.

Production, selection, circulation, use, breakage, discard, and depositional filtering remain possible contributors to the observed profiles, but no one of these is inferred directly from the matrices.

Location in revised manuscript: pp. 4 and 9–14, especially lines 73–99 and 207–349; pp. 24–26, lines 566–643.

Comment 6

“The archaeological context should be presented more directly. Wacheqsa, the five analytical units and their grouping into earlier and later assemblages should be explained before the detailed analytical discussion. Urabarroid and Janabarroid should be defined at first use.”

Response

I reorganized the opening of the paper around this request.

The Introduction now begins by locating Wacheqsa immediately north of the monumental core of Chavín de Huántar, names the five analytical contexts, and defines the Urabarroid and Janabarroid aggregates before introducing the statistical problem.

The archaeological section was also expanded. The revised manuscript distinguishes Early Platforms, Water Flood, Stone Rooms, Midden, and Late Platforms by stratigraphic position, formation process, excavation context, and material inventory.

A new schematic Figure 1 provides the site and excavation orientation, while Figure 2 shows the documented exposures associated with the analytical units. Table 1 summarizes the archaeological evidence relevant to each context.

Location in revised manuscript: pp. 3–9, lines 42–223.

Comment 7

“The evolutionary discussion can also be shortened unless the author can link it to predictions that are actually tested by the present data.”

Response

I shortened and repositioned that discussion.

The revised paper does not infer conformity, prestige bias, neutral drift, selection, centralized production, or particular learning pathways from the observed frequency distributions.

Where evolutionary concepts remain, they are used only to identify hypotheses that would require additional evidence. The Discussion now specifies the kinds of evidence needed to distinguish such mechanisms, including vessel-equivalent observations and refits, technological and toolmark evidence, compositional provenance, use-wear or residue data, and finer event-level formation histories.

The paper’s contribution is therefore framed as an archaeological analysis of assemblage composition and association structure, not as a test of a specific evolutionary mechanism.

Location in revised manuscript: pp. 23–25, lines 540–618, particularly the section “What the data do not establish.”

Comment 8 — Clarity and presentation

“The writing needs significant reworking for clarity…”

Response

I revised the manuscript throughout for clarity and reduced several sources of ambiguity identified in the review. I removed the earlier formulation “continuity with redistribution,” defined the analytical terminology at first use, reorganized the archaeological context before the quantitative analysis, shortened the excavation and evolutionary discussions, and separated empirical results from possible behavioral explanations. The revised manuscript also distinguishes consistently among ware composition, technique composition, joint composition, and ware–technique association.

Reviewer 1

Reviewer 1’s report was written as a continuous critique rather than as numbered comments. I respond below to its main substantive points using the reviewer’s own wording.

Comment 1

“It is not clear to me what the goal of the MS is. Is it a methodological contribution? Or one that hopes to contribute to Chavin studies and the anthropological meaning of changes in pottery making practices…? Where are the humans?”

Response

This criticism led me to reorganize the paper.

The revised manuscript is not presented primarily as a demonstration of statistical methods. It asks an archaeological question: what actually changed in the Wacheqsa ceramic assemblages, and can that change be reduced to differences in ware abundance or decoration frequency alone?

The Discussion now begins with “What changed archaeologically at Wacheqsa?” The analysis shows categorical persistence in much of the ware repertoire together with changes in ware abundance, decorative emphasis, and ware–technique association.

I also expanded the treatment of the contexts in which those assemblages formed. Early Platforms are discussed in relation to localized domestic/provisioning activity; Stone Rooms in relation to segmented architectural space and a broader material inventory; Midden in relation to recurrent collective consumption, cleaning, and secondary disposal; and Late Platforms as closure/reorganization fills.

At the same time, I have avoided assigning the observed profiles directly to particular groups of people or institutions where the data do not support that inference.

Location in revised manuscript: pp. 20–24, lines 465–591.

Comment 2

The reviewer questioned the earlier formulation of “continuity with redistribution” and asked what “redistribution” meant archaeologically.

Response

I removed that formulation.

The revised manuscript now separates several different empirical observations instead of combining them under “redistribution”.

First, nine of the eleven ware categories represented across the two aggregates occur in both. That is categorical persistence.

Second, the relative abundance of those wares changes.

Third, decorative-technique frequencies change substantially, particularly through the increase in stamping/impression and decline in incision/modeling.

Fourth, the association linking techniques to continuing wares also changes.

The resulting interpretation is now phrased as substantial change within a largely persistent repertoire, or more specifically as categorical persistence accompanied by compositional and relational change.

Location in revised manuscript: Abstract, p. 2; Discussion, pp. 20–21, lines 465–513; Conclusions, pp. 25–26, lines 592–643.

Comment 3

“To me, the analysis presented amounts to an exercise in circular reasoning…”

Response

I revised the manuscript specifically to separate the contexts being compared from the historical typological labels.

The Wacheqsa analytical units were established in the original post-excavation work from stratigraphy, deposit geometry, sediment matrix, architectural association, spatial continuity, and formation history. Their principal stratigraphic ordering does not derive from the standardized ware–decoration frequency analysis reported here.

Urabarroid and Janabarroid are retained only as descriptive affinity labels attached to those context aggregates.

The paper also no longer treats Burger’s historical types as independent variables. Reconstructing those types from ware, finish, form, and decoration and then testing them using overlapping attributes would itself create the circularity raised by the reviewer.

The empirical comparison instead uses recorded ware categories, broad techniques, and their joint distribution.

Location in revised manuscript: pp. 3–8, lines 42–206; pp. 10–11, lines 224–275; pp. 24–25, lines 566–618.

Comment 4

The reviewer argued that demonstrating only that Urabarriu and Janabarriu share some attributes would have little archaeological value because this is already implicit in typological classification.

Response

I agree with that criticism, and the revised manuscript no longer treats shared categories by themselves as the principal result.

The direct marginal comparisons show what would be learned from wares alone and techniques alone. The additional association model then asks a different question: whether the same decorative techniques occur on the same wares in the same proportions once both marginal distributions are allowed to differ.

The rejection of homogeneous association demonstrates that the change is relational as well as compositional. Familiar ware categories persist, but their relationships with decorative techniques differ between the earlier and later context sets.

That is the specific information not supplied by simply observing that the named assemblages contain both similarities and differences.

Location in revised manuscript: pp. 13–16, lines 301–399; Discussion, p. 21, lines 488–513.

Comment 5

“If so, why not present distribution maps, stratigraphic sections and more data on the archaeological contexts themselves?”

Response

I added considerably more spatial and contextual information.

Figure 1 now provides a schematic site and excavation map locating Wacheqsa relative to the monumental core and neighboring sectors. Figure 2 maps the documented excavation exposures in which the analytical units occur, including the WQ-7 systematic-sampling frame.

The text now explains that apparent spatial overlap among analytical units reflects superposition in three-dimensional stratigraphy rather than mutually exclusive surface zones. The original context-level Harris matrix is also provided as S1 Fig.

Table 1 was expanded to summarize formation and stratigraphy, excavated inventory, and contextual interpretation for each analytical unit.

Location in revised manuscript: pp. 3 and 5–9, lines 42–72 and 100–223; Supporting Information caption on p. 30.

Comment 6

“What seems of value might be to try to understand how and why ceramic technologies and decorative tastes change over time…”

Response

The revised Discussion addresses the archaeological implications of the observed changes but stops short of attributing them to causes the present data cannot discriminate.

The assemblages show a marked increase in stamping/impression across all three later contexts, but the later contexts differ in their ware–technique configurations. This indicates a shared broad decorative orientation without a single homogeneous Janabarroid profile.

Possible explanations include production choices, visual or tactile preferences, vessel selection, circulation, use setting, and depositional selection. The present sherd-count matrices cannot distinguish among these alternatives.

The manuscript therefore identifies the archaeological pattern first and defines the additional evidence needed for causal interpretation rather than selecting a mechanism from the frequency data alone.

Location in revised manuscript: pp. 22–24, lines 514–591.

Comment 7

The reviewer recommended either presenting enough contextual and ceramic evidence to contribute directly to Chavín studies or reframing the paper explicitly as a methodological contribution.

Response

I chose the first route.

The revised manuscript is framed as an archaeological contribution centered on Wacheqsa and Chavín rather than as a methods paper. Statistical procedures are used to answer the archaeological question and are not presented as the primary contribution in themselves.

The Discussion now considers the ceramic change against the independently documented architectural reorganization of the monumental core. The modeled Wacheqsa transition at approximately 878–806 cal BCE broadly overlaps the onset of Black and White Stage reorganization, but the paper explicitly states that this correspondence does not establish event-level synchrony or common causation.

This is presented as a site-wide hypothesis requiring independently dated assemblages from other sectors, not as a causal conclusion.

Location in revised manuscript: pp. 23–24, lines 540–591; Conclusions, pp. 25–26, lines 592–643.

Reviewer 2

Comment 1

“My main concern is with the paper’s contrast between decoration and ceramic types… What it does not yet show is that looking at decoration produces more, or different, archaeological interpretation than looking at types or wares alone.”

The reviewer further asked: “Using the same count matrices, show a ware/type-only analysis, a decoration-only analysis, and the joint ware-by-decoration analysis. Then say what conclusion changes when decoration is included.”

Response

I made this the central analytical revision. The earlier and later assemblages are now compared on exactly the same nine-ware × six-technique support at three levels: ware composition (H = 0.430), technique composition (H = 0.588), and joint ware × technique composition (H = 0.734). Because joint H includes both marginal and association differences, I do not treat the difference among these distances as an association effect. A homogeneous-association model therefore tests the relational question directly after aggregate-specific ware and technique margins are fitted. A common ware–technique association is rejected (G² = 118.59; pboot = 0.0001). The added archaeological result is thus not simply that decoration changes, but that decoration is distributed differently among continuing wares after changes in both margins are accounted for.

The manuscript now states explicitly what each analytical level contributes: ware-only analysis shows persistence with shifts in relative ware abundance; technique-only analysis shows the strong movement away from incision/modeling toward stamping/impression; the joint analysis shows that this decorative change is not distributed uniformly across the continuing wares.

Location in revised manuscript: pp. 3–4, lines 42–99; pp. 13–16, lines 301–399; p. 21, lines 488–513; pp. 25–26, lines 592–643.

Comment 2

“The paper never defines clearly what it means by a ceramic ‘type’ in relation to a ware and a decoration. This is important because archaeological types may already include decoration, while the ware labels in this paper partly include surface finish.”

Response

The terminology has been rewritten. “Ware” now refers to the recorded macroscopic color-plus-surface-finish categories; “detailed decoration” to the standardized decorative or surface-treatment label assigned to a sherd; “broad technique” to the higher-order analytical grouping; and “type” only to historically published composite constructs such as Burger’s Urabarriu, Chakinani, and Janabarriu categories.

I also explain why a reconstructed Burger-type variable would not provide an independent comparator: those types combine attributes that overlap with the variables analyzed here. Reconstructing the types from the same attributes and then testing them against those attributes would reproduce the circularity raised during review. The recorded ware margin is therefore used as the non-decoration compositional baseline.

Location in revised manuscript: pp. 3–4, lines 42–99; pp. 10–11, lines 224–275.

Comment 3

“The Wacheqsa sequence is at once too detailed and not detailed enough. I wanted a clearer high-level orientation before the excavation details.”

Response

I reorganized the opening archaeological section. The Introduction now first locates Wacheqsa relative to the monumental core of Chavín de Huántar, identifies the five stratigraphically differentiated depositional contexts, explains their grouping into earlier and later aggregates, and states immediately that the comparison is neither a five-stage sequence nor a population estimate.

Figure 1 now provides a schematic site and excavation orientation, Figure 2 shows the documented analytical-unit exposures, and Table 1 concentrates the stratigraphic, formation, inventory, and contextual information needed for the comparison.

Location in revised manuscript: pp. 3–9, lines 42–223.

Comment 4

“Urabarroid and Janabarroid need to be defined before their first use… A short definition at first use would solve this problem.”

Response

They are now defined in the opening paragraph. Urabarroid denotes Early Platforms + Water Flood; Janabarroid denotes Stone Rooms + Midden + Late Platforms. The -oid suffix indicates ceramic affinity within pre-existing archaeological context sets and does not designate an independently defined phase or ceramic type. The same distinction is repeated in the Methods when the analytical units and aggregate labels are constructed.

Location in revised manuscript: p. 3, lines 42–72; pp. 6–7, lines 130–182.

Comment 5

“Much of the excavation volume, artifact-density, and inventory information can move to Table 1 or the supplement. The prose should keep only what is needed for the comparison…”

Response

I condensed the excavation narrative and moved the comparative inventory information into Table 1. The prose now concentrates on stratigraphic position and formation history: the water-laid character of Water Flood, the cumulative-refuse character of Midden, the room-and-alley context of Stone Rooms, and the closure/reorganization character of Late Platforms. The 1945 landslide is mentioned only to explain why interpretation depends on excavated stratigraphy.

Location in revised manuscript: pp. 5–9, lines 100–223; Table 1.

Comment 6

“I would also add a compact table in the main text listing the nine shared ware codes and full names, along with the six broad decorative techniques used in the principal comparison.”

Response

Table 2 was added for exactly this purpose. It lists the nine shared ware categories and the six broad techniques used in the principal 9 × 6 common-support analysis, and it states which aggregate-specific categories are excluded from that comparison and why. The complete lexical concordance remains in S1 Table.

Location in revised manuscript: pp. 10–11, lines 224–275; Table 2.

Comment 7

“I am less convinced that the evolutionary framework needs so much space… the evolutionary material should be shorter and kept as a statement about questions for future work.”

Response

I shortened and repositioned that discussion. The revised manuscript does not infer learning, drift, selection, conformity, prestige bias, centralized production, or production organization from the observed frequency distributions. Evolutionary concepts are retained only to identify hypotheses that would require independent vessel-level, technological, compositional, spatial, and formation-history evidence.

Location in revised manuscript: pp. 23–25, lines 540–618, especially “What the data do not establish.

Comment 8

Reviewer 2 reported that the reproducibility workflow ran successfully and that the validation checks, fixed seeds, session information, reference results, generated figures, and checksum manifest made the numerical analysis easier to assess.

Response

I retained this structure and updated the release to analysis package v12.4 under R 4.5.2. The authoritative workflow now begins from the five analytical-unit matrices, verifies their cellwise aggregation, reproduces the reported analyses and figures, records session information, and runs output validation. From the project root, the complete workflow is executed with:

source("R/00_run_all.R")

Location in revised manuscript: p. 15, lines 350–374; Data Availability, p. 27, lines 644–667.

Comment 9

“The OSF project would benefit from a better public front page. It currently has only a one-sentence description, no wiki, no visible license, and no immutable registration… The OSF overview should also identify the manuscript version, explain the difference between the OSF title and the submitted title, map R.zip, the supplementary tables, and the figures to the paper, and give the one-command reproduction route.”

Response

The OSF record has now been finalized around analysis package v12.4. The public documentation identifies the manuscript and analysis-package version, describes the repository structure, maps the analysis archive, supplementary tables, and figure outputs to the manuscript, and provides the one-command reproduction route. Licensing for the analytical code and for the data/supporting materials is stated explicitly.

Additional journal requirements

I have also addressed the technical requirements listed in the decision letter.

Journal requirement 1 — PLOS ONE style and file naming

The revised manuscript and accompanying files have been formatted and named in accordance with the PLOS ONE submission requirements. A clean revised manuscript and a version showing tracked changes are included with the resubmission.

Journal requirement 2 — Ethics statement

The permits and ethics statement now appears only in the Methods. It states that the Wacheqsa fieldwork was conducted under authorizations issued by the Instituto Nacional de Cultura del Perú in 2003, 2004, and 2005 and that all necessary permits were obtained in compliance with the relevant regulations.

Journal requirement 3 — Funding Information and Financial Disclosure

The Funding Information and Financial Disclosure entries have been reconciled in Editorial Manager. The corrected disclosure is: “The author received support for Wacheqsa fieldwork and doctoral research from a Stanford University School of Humanities and Sciences Graduate Research Opportunity Grant, the Stanford Department of Anthropological Sciences, the Stanford Center for Latin American Studies, and the Stanford Archaeology Center. Grant numbers were not assigned. The funders had no role in study design, data analysis, decision to publish, or preparation of the manuscript.

Journal requirements 4–5 — Figure copyright and permissions

The copyrighted/proprietary image content identified in the original submission has been removed or replaced. Figure 1 is a newly prepared schematic site map based on project base maps and contains no proprietary map or satellite imagery. Figure 2 derives from the author’s project CAD excavation model. Figure 3 is a newly prepared composite from original Wacheqsa project specimen drawings rather than a reproduction of a previously published third-party figure. The revised Figures 1–3 therefore do not reproduce the proprietary cartographic or third-party published figure content flagged in the original submission.

Journal requirement 6 — Supporting Information files

All Supporting Information files cited in the manuscript—S1–S3 Figs and S1–S5 Tables—are included in the Supporting Information package uploaded with the revision.

The review led me to narrow the paper’s claims.

The revised manuscript does not argue that Urabarroid and Janabarroid are two homogeneous ceramic populations, that the Wacheqsa data validate Burger’s site-wide phase sequence, that Chakinani has been disproved, or that a particular social or evolutionary mechanism caused the observed pattern.

The result supported by the present data is more specific. Across the stratigraphically ordered Wacheqsa assemblages, ware composition changes, decorative-technique composition changes, and ware–technique association changes as well, while much of the coded ware repertoire persists. The later contexts also remain internally differentiated.

I believe that this formulation better reflects both the archaeological evidence and the limits of the analytical sample.

Thank you again for the careful reviews and for the opportunity to revise the manuscript.

Christian Mesía-Montenegro

Attachment Submitted filename: Response to the Academic Editor and Reviewers.docx

# Peer review: Decision Letter 1

**DOI:** 10.1371/journal.pone.0361889.r003

**Authors:** Przemysław Mroczek

12 Oct 2026

Dear Dr. Mesia-Montenegro,

Thank you for submitting your manuscript to PLOS One. After careful consideration, we feel that it has merit but does not fully meet PLOS One’s publication criteria as it currently stands. Therefore, we invite you to submit a revised version of the manuscript that addresses the points raised during the review process.

Although Reviewer 2 recommends acceptance, my assessment identified unresolved problems with the documentation of the Bayesian chronology, the interpretation of uneven archaeological samples, specimen identification, and inconsistencies in the references and funding declaration. These matters must be addressed to meet PLOS ONE’s requirements for scientific rigour and verifiable reporting, while the suggested change to the title is optional; my detailed comments and justification for Major Revision are provided below.

Please submit your revised manuscript by Nov 26 2026 11:59PM. If you will need more time than this to complete your revisions, please reply to this message or contact the journal office at plosone@plos.org. When you're ready to submit your revision, log on to https://www.editorialmanager.com/pone/ and select the 'Submissions Needing Revision' folder to locate your manuscript file.

- A letter that responds to each point raised by the academic editor and reviewer(s). You should upload this letter as a separate file labeled 'Response to Reviewers'.
- A marked-up copy of your manuscript that highlights changes made to the original version. You should upload this as a separate file labeled 'Revised Manuscript with Track Changes'.
- An unmarked version of your revised paper without tracked changes. You should upload this as a separate file labeled 'Manuscript'.

If you would like to make changes to your financial disclosure, please include your updated statement in your cover letter. Guidelines for resubmitting your figure files are available below the reviewer comments at the end of this letter.

If applicable, we recommend that you deposit your laboratory protocols in protocols.io to enhance the reproducibility of your results. Protocols.io assigns your protocol its own identifier (DOI) so that it can be cited independently in the future. For instructions see: https://journals.plos.org/plosone/s/submission-guidelines#loc-laboratory-protocols. Additionally, PLOS One offers an option for publishing peer-reviewed Lab Protocol articles, which describe protocols hosted on protocols.io. Read more information on sharing protocols at https://plos.org/protocols?utm_medium=editorial-email&utm_source=authorletters&utm_campaign=protocols.

As the corresponding author, your ORCID iD is verified in the submission system and will appear in the published article. PLOS supports the use of ORCID, and we encourage all coauthors to register for an ORCID iD and use it as well. Please encourage your coauthors to verify their ORCID iD within the submission system before final acceptance, as unverified ORCID iDs will not appear in the published article. *Only*  the individual author can complete the verification step; PLOS staff *cannot*  verify ORCID iDs on behalf of authors.

We look forward to receiving your revised manuscript.

Kind regards,

Przemysław Mroczek, Dr. hab.

Academic Editor

PLOS One

Journal Requirements:

If the reviewer comments include a recommendation to cite specific previously published works, please review and evaluate these publications to determine whether they are relevant and should be cited. There is no requirement to cite these works unless the editor has indicated otherwise.

Additional Editor Comments:

The author has addressed many of the criticisms raised in the first round. The purpose of this further revision is to improve the scientific quality of the manuscript before publication, particularly the documentation of its chronology, the treatment of archaeological contexts and samples, and the traceability of the evidence. Reviewer 2 now recommends acceptance, and the revised statistical analysis is easier to follow. I nevertheless found several problems that need to be resolved. Some concern the interpretation of the results; others are errors or gaps in the accompanying documentation. I recommend Major Revision for the reasons set out below.

Comments to the Author

1/

On page 7 (lines 157-160), the transition between the Urabarroid and Janabarroid aggregates is dated to 878–806 cal BCE, and the end of the Janabarroid sequence to 760–581 cal BCE, both at 68.3% probability. The cited source is reference [15], Mesía-Montenegro and Weber (2024), Starch Analyses on Culinary Equipment from Chavin de Huantar. That paper deals principally with microbotanical evidence. As the citation stands, I cannot establish where the reported Bayesian age ranges come from.

Please identify the source of these estimates precisely: the published model, table or figure, and the archaeological and radiocarbon evidence on which it rests. If reference [15] contains the model, give its exact location. If the estimates were published elsewhere, cite that source. Reference [19] on a Bayesian reassessment of Andean Formative chronology, appears in the bibliography but is not cited in the text; please check whether it is relevant rather than simply replacing one citation with another.

A compact table of the radiocarbon determinations used to constrain the Wacheqsa sequence, their contexts and the calibrated or modelled ranges would help the reader assess this part of the argument. If the model is already fully published, the table could instead direct the reader to the original data and model specification. The problem also affects the discussion on pages 23–24 (lines 561–580), where the ceramic transition is compared with the architectural reorganisation of the monumental centre. That comparison may be reasonable, but its chronological basis must be traceable.

2/

The five archaeological units are described more fully, and the manuscript now distinguishes the stratigraphic context sets from the Urabarroid and Janabarroid affinity labels. This distinction matters, but it does not resolve the problem of comparing deposits with different origins and functions. Early Platforms includes floors, platform surfaces and fills; Water Flood contains water-laid material; Stone Rooms includes rooms, alleys and cleaning deposits; Midden is accumulated refuse; and Late Platforms consists of construction and closure-related fills (pp. 5–9, especially lines 135–148 and 197–210).

These contexts are not equivalent samples of successive occupation stages. Their ceramic differences may reflect elapsed time, but also vessel use, disposal, reworking and the selection of material entering each deposit. Please separate the evidence for stratigraphic order from the interpretation of change in ceramic practice. Where does superposition establish an earlier–later relationship? Where is the argument dependent on ceramic affinities or a wider chronological model?. The Discussion should say more clearly which conclusions concern differences between particular contexts and which can reasonably be described as changes through time.

3/

The imbalance in the sample needs to remain central to the interpretation. Early Platforms accounts for 330 of the 352 observations in the earlier aggregate (93.8%); Midden supplies 691 of the 956 observations in the later aggregate (72.3%). Water Flood has only 22 observations and Late Platforms 50 (pp. 9 and 11).

The analysis excluding Midden is useful, and the principal numerical difference persists. But this does not turn the remaining contexts into comparable chronological samples. Early Platforms still dominates the earlier aggregate, while the later units have different activity and formation histories. I would therefore like to see the context-to-context comparisons in Tables 4 and 5 used more directly in the Discussion, particularly the comparisons between Early Platforms and each of the three later units. What conclusions remain when the pooled aggregates are set aside?

Water Flood needs special caution. The reported 27.3% stamping/impression represents six sherds out of 22. Combined with its water-related formation history, that small count cannot establish a transitional ceramic stage. The manuscript recognises this limitation, but it should not be lost when the broader sequence is discussed.

4/

The full analytical corpus comprises 1,308 decorated or surface-treated sherd observations. The principal 9-ware × 6-technique comparison uses 334 observations from Urabarroid and 822 from Janabarroid. Thus 18 of 352 earlier observations (5.1%) and 134 of 956 later observations (14.0%) are excluded.

Pages 10–11 (lines 225-271) explain why the principal comparison is restricted to shared categories, and the manuscript includes broader-support sensitivity analyses. What is missing is a straightforward account of the different proportions excluded from the two aggregates. Please show how the 1,308 observations were reduced to the common-support dataset, distinguishing exclusions due to aggregate-specific ware categories from those due to decorative-technique categories. A small table would suffice.

The effect of these decisions should also be discussed with the numerical results in view. The shared-support joint Hellinger distance is H = 0.734, compared with H = 0.763 on union support. The difference is not in itself evidence of an invalid analysis, but readers need to know how the reported distance changes when the excluded categories are retained.

5/

On page 10 (lines 242-252), the author explains that each sherd received one detailed decoration or surface-treatment label, although an individual vessel could carry several treatments. The original records do not preserve the rule used when more than one treatment was present. Nor do they document the handling of eroded or doubtful specimens or any checks of agreement within or between observers.

This matters for the main result. The study tests how ware categories are associated with decorative techniques; assigning a multiply decorated sherd to only one class may alter the frequencies in the contingency table. I am not asking for the assemblage to be reclassified. Please state what can still be established about the original coding procedure, what cannot be recovered, and how the missing information might affect the association analysis. The implications deserve a place in the Discussion as well as in the Methods.

6/

The added homogeneous-association model responds to an important criticism from the first review round. The distinctions between ware margins, technique margins, joint composition and association heterogeneity are now clear. The remaining difficulty is the archaeological meaning attached to the statistical test.

The bootstrap result (p_boot = 0.0001) is evidence against the fitted common-association model under its assumptions. It cannot, by itself, show that the observed differences arose through chronological change, and it does not account for the possibility that several sherds came from the same vessel. Please keep these limits explicit wherever the model is used to support an archaeological conclusion.

The discussion would also benefit from more attention to the actual ware–technique combinations. For instance, page 20 (lines 475–478) reports pronounced changes in incision and stamping/impression within Gray Polished, Black Polished and Brown Polished wares. Those examples show the reader what changed in the material. They should carry more of the interpretation, alongside the model results, instead of leaving the statistical rejection as the main point.

7/

Figure 3 illustrates fragments assigned to the two aggregates and identifies their ware categories and proveniences. None of the illustrated sherds, however, has an individual specimen, excavation catalogue or museum inventory number. The statement that the collection is held by the Museo Nacional de Chavín, Ancash, Peru, does not allow a reader to identify the objects shown.

Please add catalogue or inventory identifiers to the caption or a supplementary concordance, together with enough information to link each drawing to its excavation context and the curated collection. This would also help meet the journal's requirements for documenting archaeological specimens. If the older records do not permit individual identification, explain what identifiers survive and how the illustrated material could be located or examined.

8/

The Permits and Ethics section (p. 15, lines 357-366) identifies the Instituto Nacional de Cultura del Perú as the authority that authorised the fieldwork in 2003, 2004 and 2005. It does not provide permit or authorisation numbers.

Please supply the reference numbers where available. If the records cannot be recovered or numbers were not assigned, state that plainly. This is a request for complete documentation of the earlier fieldwork, not a suggestion that the excavations were unauthorised.

9/

The bibliography needs a full check after the revision. PLOS ONE numbers references in order of first citation, but the first citation in the Introduction is [17,18,20] (p. 3, lines 67-68). The subsequent numbering is also out of sequence. The list appears to retain numbers from an earlier version of the paper.

References [1], [2], [3], [4], [6], [7] and [19] do not appear to be cited in the clean manuscript. They should either be cited where they support a specific statement or removed. Please do not retain references merely because they are relevant to the general subject.

I also found errors or gaps in individual records. The title of Mesía-Montenegro (2014), reference [13], omits Tardío from Período Formativo Tardío. Reference [16] cites Tello's 1940 archival material by bundle and notebook, but the location of the holding archive and the means of consulting it need to be made clear. If the source cannot be accessed by other researchers, an accessible supporting reference may be needed. Check titles, page ranges, DOIs and other publication details throughout, as well as the correspondence between citations and the reference list.

Data availability and reproducibility

The revised manuscript refers to the R 4.5.2 analysis package, version 12.4, deposited in OSF under DOI 10.17605/OSF.IO/KX9BT (p. 15, lines 351–355; p. 27, lines 646–650). Please confirm that this deposit is the final version used for the revised tables and figures. It should contain the five unit-level matrices, classification concordance, scripts, generated outputs and the materials needed to check the reported results.

The documented command source("R/00_run_all.R") should reproduce the analyses from those archived inputs. If the workflow has already been run and validated against the final manuscript, please state this in the response letter, giving the package version and the result of that validation. I am asking for confirmation of consistency between the paper and the archive, not asserting that the repository is incomplete.

Funding disclosure

The funding information is inconsistent across the submitted documents. The Financial Disclosure field names Stanford University, National Geographic and the Global Heritage Fund. The revised cover letter and response to reviewers instead list four Stanford University units and programmes. The author reports that the entries have been reconciled, but both versions are still present in the PDF supplied for review.

Please establish whether National Geographic and the Global Heritage Fund contributed to the work reported in this article. The final declaration in the submission system should identify all relevant funders and agree with the submission documents. Any correction to the system entry should be made with the journal office.

Title

The title identifies the site and the subject adequately, but Ceramic change followed by Changing associations is repetitive. The singular assemblage also sits awkwardly with an analysis that compares several depositional contexts. I suggest the shorter wording:

Changes in ware-decoration associations in ceramic assemblages from Wacheqsa, Chavín de Huántar, Peru.

This is a suggestion, not a required title. Whichever wording is chosen should describe the comparison without implying a continuous sequence that the sampled contexts cannot establish.

The author has now shown more clearly what the joint ware-decoration analysis adds to the separate frequency comparisons. I do not see a need to repeat the entire statistical analysis or undertake new excavation or laboratory work. The main outstanding questions concern whether the chronology can be checked, how far the unequal and functionally different samples support a temporal interpretation, and whether the archaeological material and analytical records can be traced adequately.

These points warrant Major Revision. Please address each comment in the response letter and give the page and line numbers of the corresponding changes. The revised text should make clear what the existing evidence supports and where the record does not permit a firmer conclusion.

[Note: HTML markup is below. Please do not edit.]

Reviewers' comments:

Reviewer's Responses to Questions

**Comments to the Author**

Reviewer #2: All comments have been addressed

**********

2. Is the manuscript technically sound, and do the data support the conclusions?

Reviewer #2: Yes

**********

3. Has the statistical analysis been performed appropriately and rigorously?

Reviewer #2: Yes

**********

4. Have the authors made all data underlying the findings in their manuscript fully available?

The PLOS Data policy

Reviewer #2: Yes

**********

5. Is the manuscript presented in an intelligible fashion and written in standard English?

Reviewer #2: Yes

**********

Reviewer #2: The revisions look great and adequately address all of my concerns. This is an excellent contribution to intrasite artifact analysis.

**********

what does this mean?). If published, this will include your full peer review and any attached files.

If you choose “no”, your identity will remain anonymous but your review may still be made public.

**Do you want your identity to be public for this peer review?**  For information about this choice, including consent withdrawal, please see our Privacy Policy

Reviewer #2: **Yes:**  Robert J. Bischoff

**********

[NOTE: If reviewer comments were submitted as an attachment file, they will be attached to this email and accessible via the submission site. Please log into your account, locate the manuscript record, and check for the action link "View Attachments". If this link does not appear, there are no attachment files.]

To ensure your figures meet our technical requirements, please review our figure guidelines: https://journals.plos.org/plosone/s/figures

You may also use PLOS’s free figure tool, NAAS, to help you prepare publication quality figures: https://journals.plos.org/plosone/s/figures#loc-tools-for-figure-preparation.

NAAS will assess whether your figures meet our technical requirements by comparing each figure against our figure specifications.

# Author comment: Author response to Decision Letter 2

**DOI:** 10.1371/journal.pone.0361889.r004

14 Oct 2026

Manuscript PONE-D-26-35517R1

Changes in ware–decoration associations in ceramic assemblages from Wacheqsa, Chavín de Huántar, Peru

Dear Dr. Mroczek,

Thank you for the detailed comments. I have clarified the chronological sources, distinguished stratigraphic relationships from contextual interpretation, and expanded the documentation of the analytical samples. Below, I respond to each point and identify the corresponding changes. The numerical results remain those of the validated version 12.4 package.

1 Bayesian chronology

Comment: The editor requests precise sources for the modeled boundaries, the radiocarbon and contextual evidence underlying the Wacheqsa chronology, and a traceable basis for comparison with architectural reorganization.

Response: I have retained the citation to Mesía-Montenegro and Weber because its published Bayesian model supplies the reported estimates. I now give the exact location: Figure 3, p. 781, and Table 1, p. 782 (revised reference [12]). The End Urabarroid–Start Janabarroid estimate is 878–806 cal BCE and End Janabarroid is 760–581 cal BCE, both at 68.3% probability. Conventional determinations and analytical-unit assignments appear in Mesía-Montenegro (2022), Table 2, p. 887 (revised reference [10]); its Figure 3 shows calibrated determinations, not modeled aggregate boundaries.

Main-text Table 2 lists the ten laboratory determinations, conventional ages and uncertainties, analytical-unit assignments, published modeled intervals, and excavation contexts. The source note identifies the publications and dissertation sections used. I have not recalibrated the dates or fitted a new Bayesian model. The text distinguishes this aggregate chronology from dates of ceramic production or changes in individual decorative practices.

I have retained the architectural comparison as a historical question. Establishing synchronicity or a shared institutional mechanism would require comparison of independently constrained model estimates and their uncertainty across sectors. The ceramic association test does not establish that synchronicity.

Changes: pp. 7–8, lines 156–170, and Table 2; p. 23, lines 540–556, “Stratigraphic order and chronological interpretation.”

2 Archaeological contexts and temporal interpretation

Comment: The editor asks that direct stratigraphic order be separated from correlations, ceramic affinities, and interpretations of change across deposits with different formation histories.

Response: I have clarified that the five analytical units were constructed before the present frequency analysis using stratigraphic, spatial, architectural, sedimentary, and depositional evidence. Their construction was independent of the standardized frequency matrices, but was not blind to all archaeological material.

I now distinguish local superposition from cross-exposure correlation. Stone Rooms overlies Early Platforms in several exposures; the relationship between WQ-4 layer 6 and the Stone Rooms network represented by WQ-8 layer 3 involves correlation between exposures. Midden overlies or cuts Water Flood in southern WQ-7. The Early Platforms–Late Platforms comparison is qualified because the deeper Late Platforms determination AA75388 was already recognized as potentially overlapping the earlier occupation. Table 7 now acknowledges this qualification.

The Discussion treats the units as distinct activity and depositional settings. It retains an archaeological interpretation of differing decorative repertoires while recognizing that elapsed time cannot be separated from activity composition, use, disposal, and reworking. A continuous or equally spaced five-stage sequence is not inferred.

Changes: pp. 6–7, lines 121–153; Table 7; pp. 22–23, lines 503–556.

3 Unequal samples and direct context comparisons

Comment: The editor requests greater interpretive use of individual context comparisons and continued caution about the small Water Flood sample.

Response: The numerical imbalance remains explicit: Early Platforms contributes 330 of 352 earlier observations (93.8%), and Midden contributes 691 of 956 later observations (72.3%). These are shares of the analytical sample, not estimates of archaeological prevalence.

Tables 6 and 7 support direct comparisons of Early Platforms with Stone Rooms, Midden, and Late Platforms. Stamping/impression comprises 6/330 observations in Early Platforms (1.8%), 117/215 in Stone Rooms (54.4%), 456/691 in Midden (66.0%), and 34/50 in Late Platforms (68.0%). Its prominence is therefore not confined to the largest refuse sample. I also discuss differences among the later joint profiles rather than treating the pooled Janabarroid aggregate as homogeneous.

The leave-Midden-out comparison checks the influence of the largest later sample; it does not make the remaining deposits functionally equivalent. Water Flood’s 27.3% stamping/impression represents six observations out of 22. This small, water-laid sample cannot establish a transitional stage. I also qualify the interpretation of the small Late Platforms sample.

Changes: p. 11, lines 267–270; Tables 6 and 7; pp. 21–22, lines 490–513.

4 Reduction to common support

Comment: The editor requests explicit accounting for the differing exclusion proportions and discussion of the shared-support and union-support distances.

Response: Table 4 now gives mutually exclusive exclusion categories. In the earlier aggregate, two observations are excluded for ware alone, fifteen for technique alone, and one for both: eighteen of 352 (5.1%), leaving 334. In the later aggregate, the corresponding counts are 67, 66, and one: 134 of 956 (14.0%), leaving 822. The text also explains the sequential filter, which removes three and 68 observations for aggregate-specific wares before excluding fifteen and 66 for technique among the retained wares.

The joint Hellinger distance is H = 0.734 on shared support, H = 0.752 with all nine broad classes retained within the shared wares, and H = 0.763 on full union support. Retaining the excluded categories modestly increases the descriptive contrast. Because the supports and renormalized profiles differ, this increase cannot be assigned as an additive contribution of particular excluded categories.

Changes: p. 11, Table 4 and lines 261–265; pp. 18–19, lines 425–450; p. 24, lines 564–567; S4 Table.

5 Original coding procedure

Comment: The editor asks what is known about the original single-label procedure, what cannot be recovered, and how those gaps could affect the association analysis.

Response: I have specified what survives: one decoration or surface-treatment label per sherd, with matrices and a concordance documenting how those labels were grouped into broad classes. Co-occurring treatments and the original rule for choosing among them are not preserved. Procedures for eroded or ambiguous specimens and observer-agreement checks are undocumented.

A treatment-priority rule could affect recorded ware–technique frequencies, even if applied consistently, when treatment co-occurrence differed among wares or contexts. Without the additional treatments or original selection rule, this effect cannot be quantified. The analysis therefore concerns recorded labels rather than the complete distribution of treatments on individual sherds. I have not retrospectively reclassified them.

Changes: p. 10, lines 234–248; p. 24, lines 558–563; S1 Table.

6 Meaning of the homogeneous association test

Comment: The editor requests explicit limits on temporal attribution and vessel independence, and greater emphasis on actual ware–technique combinations.

Response: I have clarified that model rejection does not identify an archaeological mechanism. The homogeneous-association test evaluates a common association after fitting aggregate-specific margins. Its bootstrap calibration applies to the fitted sherd-count model; it does not account for unknown clustering of fragments within vessels or reconstruct the original physical sampling design.

I now emphasize the actual combinations. Within retained Gray Polished observations, incision changes from 89.4% to 5.3%, while stamping/impression changes from 0% to 86.0%. Incision changes from 93.3% to 12.4% in Black Polished and from 80.0% to 11.2% in Brown Polished; stamping/impression changes from 4.4% to 81.1% and from 8.0% to 84.1%, respectively. These are within-ware percentages, not overall ware abundances.

The interpretation relates these combinations to documented activity settings, collective consumption, and possible movement into refuse and construction fills. A role for decoration in serving or presentation remains a contextual hypothesis. The matrices alone cannot establish that role or demonstrate rank, affiliation, ritual authority, or institutional transformation.

Changes: pp. 13–14, lines 312–336; pp. 15–19, lines 365–450; pp. 20–22, lines 468–539; p. 24, lines 558–573; Fig 5 and S5 Table.

7 Illustrated specimen identification

Comment: The editor requests specimen identifiers and links between each Figure 3 drawing, excavation context, and curated collection, or a precise explanation of the surviving documentation.

Response: Figure 3 has been updated to show the recorded excavation provenience for each of its 19 illustrated sherds, including excavation unit, level and, where applicable, sector and subunit. Panel A derives from Early Platforms; in panel B, a–c, e–g and j derive from Midden, while d, h and i derive from Stone Rooms. These locators substantially improve the traceability of the illustrations but are not unique specimen or museum inventory numbers. The archaeological collection is curated at the Museo Nacional de Chavín, Ancash, Peru. A unique drawing-to-object concordance is not preserved in the analytical archive; identifying an individual object would require checking the original drawing sheets against the collection records.

Changes: p. 10, Fig 3 and caption, lines 215–232.

8 Fieldwork authorizations

Comment: The editor requests reference numbers for the 2003–2005 authorizations.

Response: The Permits and Ethics section now gives the Instituto Nacional de Cultura del Perú authorizations: Resolución Directoral Nacional Nº 824/INC, 4 August 2003; Nº 704/INC, 23 August 2004; and Nº 902/INC, 4 July 2005. The statement also distinguishes the original fieldwork from the present analysis of previously classified material, which involved no new excavation, destructive sampling, or export.

Changes: p. 15, lines 353–362, “Permits and ethics.”

9 References

Comment: The editor requests sequential numbering, removal or appropriate citation of unused references, correction of the 2014 title, and clarification of the Tello archival source.

Response: I have renumbered the references in order of first citation. All 24 entries are cited in the text; previously unused entries have been cited where relevant or removed. The Wacheqsa Bayesian estimates are attributed to the published model that produced them.

The 2014 title now includes “Período Formativo Tardío.” I have removed the Tello archival citation. Sources for the sector’s archaeological and chronological background are cited where used.

Changes: citations throughout the manuscript; references on pp. 26–27, including revised reference [22].

10 Data availability and reproducibility

Comment: The editor requests confirmation that the final deposit is the package used for the revised outputs and that the documented command reproduces the analysis.

Response: The final analysis package, version 12.4, is deposited at DOI 10.17605/OSF.IO/KX9BT. I successfully executed source("R/00_run_all.R") from the package root and verified that the generated outputs agree with the numerical results reported in the revised tables and figures. The deposit contains the five unit-level matrices, classification concordance, scripts, generated outputs, bootstrap distributions, and execution/validation records.

Supporting Tables S1–S5 document the classification, aggregate and unit matrices, correspondence analysis, Hellinger comparisons, and homogeneous-association diagnostics. Chronological source information is in main-text Table 2, and specimen documentation is addressed in the Figure 3 caption.

Changes: p. 15, lines 346–351, “Reproducibility”; p. 25, lines 588–599, “Data availability”; supporting-information captions.

11 Funding disclosure

Comment: The editor asks whether the National Geographic Society and the Global Heritage Fund supported the reported work and requests consistency across the manuscript, cover letter, and Financial Disclosure field.

Response: The revised declaration identifies Stanford University, the National Geographic Society, and the Global Heritage Fund as supporters of the Wacheqsa fieldwork and doctoral research. It specifies Stanford’s contributing units and the Graduate Research Opportunity Grant, as well as the Global Heritage Fund’s provision of Autodesk Land and Autodesk Map software licenses.

The declaration states that no grant numbers were assigned and that the funders had no role in study design, data analysis, the decision to publish, or manuscript preparation. The same declaration appears in the revised manuscript and cover letter. The Financial Disclosure field will be checked for consistency during resubmission. If correction of the existing entry requires assistance from the journal office, I request that it be updated accordingly.

Changes: p. 25, lines 604–609, “Funding.”

12 Title

Comment: The editor suggests a shorter title describing assemblage comparisons without implying a continuous sequence.

Response: I have adopted the suggested title: “Changes in ware–decoration associations in ceramic assemblages from Wacheqsa, Chavín de Huántar, Peru.”

Change: title page.

13 Reviewer 2

Comment: Reviewer 2 considers the previous concerns addressed and recommends acceptance.

Response: I thank Reviewer 2 for the assessment and recommendation. This revision addresses the Academic Editor’s additional requests while retaining the numerical results and strengthening their archaeological interpretation.

Sincerely,

Christian Mesía-Montenegro

Attachment Submitted filename: Response to Editor.docx

# Editor report: Decision Letter 2

**DOI:** 10.1371/journal.pone.0361889.r005

**Authors:** Przemysław Mroczek

19 Oct 2026

Changes in ware–decoration associations in ceramic assemblages from Wacheqsa, Chavín de Huántar, Peru

PONE-D-26-35517R2

Dear Dr. Mesia-Montenegro,

We’re pleased to inform you that your manuscript has been judged scientifically suitable for publication and will be formally accepted for publication once it meets all outstanding technical requirements.

Within one week, you’ll receive an e-mail detailing the required amendments. When these have been addressed, you’ll receive a formal acceptance letter and your manuscript will be scheduled for publication.

An invoice will be generated when your article is formally accepted. Please note, if your institution has a publishing partnership with PLOS and your article meets the relevant criteria, all or part of your publication costs will be covered. Please make sure your user information is up-to-date by logging into Editorial Manager at Editorial Manager® and clicking the ‘Update My Information' link at the top of the page. For questions related to billing, please contact billing support.

If your institution or institutions have a press office, please notify them about your upcoming paper to help maximize its impact. If they’ll be preparing press materials, please inform our press team as soon as possible -- no later than 48 hours after receiving the formal acceptance. Your manuscript will remain under strict press embargo until 2 pm Eastern Time on the date of publication. For more information, please contact onepress@plos.org.

Kind regards,

Przemysław Mroczek, Dr. hab.

Academic Editor

PLOS One

Additional Editor Comments (optional):

The author has satisfactorily addressed the editorial and reviewers’ comments.

The revised manuscript demonstrates improved methodological clarity, transparency, and a more balanced interpretation of the results. The limitations of the study are appropriately acknowledged, and the supporting data are available. I consider the manuscript to meet the publication criteria of PLOS ONE and recommend its acceptance.

Reviewers' comments:

# Editor report: Acceptance letter

**DOI:** 10.1371/journal.pone.0361889.r006

**Authors:** Przemysław Mroczek

PONE-D-26-35517R2

PLOS One

Dear Dr. Mesia-Montenegro,

I'm pleased to inform you that your manuscript has been deemed suitable for publication in PLOS One. Congratulations! Your manuscript is now being handed over to our production team.

At this stage, our production department will prepare your paper for publication. This includes ensuring the following:

* All references, tables, and figures are properly cited

* All relevant supporting information is included in the manuscript submission,

* There are no issues that prevent the paper from being properly typeset

You will receive further instructions from the production team, including instructions on how to review your proof when it is ready. Please keep in mind that we are working through a large volume of accepted articles, so please give us a few days to review your paper and let you know the next and final steps.

Lastly, if your institution or institutions have a press office, please let them know about your upcoming paper now to help maximize its impact. If they'll be preparing press materials, please inform our press team within the next 48 hours. Your manuscript will remain under strict press embargo until 2 pm Eastern Time on the date of publication. For more information, please contact onepress@plos.org.

You will receive an invoice from PLOS for your publication fee after your manuscript has reached the completed accept phase. If you receive an email requesting payment before acceptance or for any other service, this may be a phishing scheme. Learn how to identify phishing emails and protect your accounts at https://explore.plos.org/phishing.

If we can help with anything else, please email us at customercare@plos.org.

Thank you for submitting your work to PLOS One and supporting open access.

Kind regards,

PLOS One Editorial Office Staff

on behalf of

Dr. hab. Przemysław Mroczek

Academic Editor

PLOS One
